# Loss of Usp9x disrupts cell adhesion, and components of the Wnt and Notch signaling pathways in neural progenitors

**DOI:** 10.1038/s41598-017-05451-5

**Published:** 2017-08-14

**Authors:** Susitha Premarathne, Mariyam Murtaza, Nicholas Matigian, Lachlan A. Jolly, Stephen A. Wood

**Affiliations:** 1 Eskitis Institute for Drug Discovery, Griffith University, Nathan Brisbane 4111, Queensland, Australia; 2 Robinson Institute, School of Paediatrics and Reproductive Health, University of Adelaide, Adelaide 5005, South Australia, Australia; 3 The University of Queensland Diamantina Institute, Translational Research Institute, Woolloongabba Brisbane 4102, Queensland, Australia

## Abstract

Development of neural progenitors depends upon the coordination of appropriate intrinsic responses to extrinsic signalling pathways. Here we show the deubiquitylating enzyme, Usp9x regulates components of both intrinsic and extrinsic fate determinants. *Nestin*-*cre* mediated ablation of Usp9x from embryonic neural progenitors *in vivo* resulted in a transient disruption of cell adhesion and apical-basal polarity and, an increased number and ectopic localisation of intermediate neural progenitors. In contrast to other adhesion and polarity proteins, levels of β-catenin protein, especially S33/S37/T41 phospho-β-catenin, were markedly increased in *Usp9x* ^−/*Y*^ embryonic cortices. Loss of Usp9x altered composition of the β-catenin destruction complex possibly impeding degradation of S33/S37/T41 phospho-β-catenin. Pathway analysis of transcriptomic data identified Wnt signalling as significantly affected in *Usp9x* ^−/*Y*^ embryonic brains. Depletion of Usp9x in cultured human neural progenitors resulted in Wnt-reporter activation. Usp9x also regulated components of the Notch signalling pathway. Usp9x co-localized and associated with both Itch and Numb in embryonic neocortices. Loss of Usp9x led to decreased Itch and Numb levels, and a concomitant increase in levels of the Notch intracellular domain as well as, increased expression of the Notch target gene Hes5. Therefore Usp9x modulates and potentially coordinates multiple fate determinants in neural progenitors.

## Introduction

Neural progenitors (NPs) are the founding cell population driving brain development. Therefore, cell fate decisions taken by NPs must balance their differentiation into neuronal and glial lineages, with self-renewal thereby facilitating ongoing growth. The fate of a NP is temporally and spatially controlled by the simultaneous exposure to multiple extrinsic signalling pathways to which NPs need to coordinate appropriate intrinsic responses^1^. Post-translational protein modifications play critical roles in mediating and dynamically integrating signalling pathways. Although the role of phosphorylation in signal transduction is well established it is clear that ubiquitylation/deubiquitylation also play critical roles in both NP fate and signal pathway regulation^2^. In the current study we focused on the role of deubiquitylating enzyme (DUB), ubiquitin specific peptidase 9 located in the X-chromosome (Usp9x) in the regulation of NP function in the mouse neocortex.

Usp9x is ideally situated to integrate extrinsic signals with intrinsic responses in NPs. *Usp9x* is highly expressed in both embryonic and adult NPs^3–5^. Conditional deletion of *Usp9x* from mouse NPs results in perinatal lethality and disrupts the cytoarchitecture of the ventricular and sub ventricular zones in late stage embryos^6^. Conversely overexpression of *Usp9x* in mouse embryonic stem cell-derived NPs significantly increased their polarity and self-renewal without affecting their differentiation capacity^3^. Although these studies demonstrate the importance of Usp9x in NP fate specification, the underlying molecular mechanism(s) remains to be elucidated. To date more than 35 proteins have been reported as Usp9x substrates, many of which are components of intrinsic and extrinsic signalling pathways known to regulate NP function^5^. However, as these Usp9x-substrate interactions were elucidated in diverse biological systems, and are often cell context specific, further characterisation of Usp9x’s role in NPs is required.

Cell adhesion and apical-basal polarity are key intrinsic regulators of NP fate^1^ and Usp9x regulates both in a number of polarised cells. Adherens junctions (AJ) are the predominant cell-cell adhesive structures in NPs and, Usp9x substrates AF-6^7^ and EFA6^8^ are key functional adhesion components. In addition Usp9x has been proposed to promote the trafficking of cadherin-catenin heterodimers during *de novo* AJ formation of T84 epithelial cells^9^. Deubiquitylation by Usp9x also activates the polarity regulatory proteins AMP-activated protein kinase related kinesin proteins NUAK1 and MARK4 in polarised epithelia^10, 11^. Functionally, cellular polarity was up regulated with modest overexpression of *Usp9x* in embryonic stem cell-derived NPs^3^.

Usp9x also regulates components of the Notch and Wnt signalling pathways, which are prominent extrinsic signalling pathways specifying NP fate^12^. The best-characterised role of Notch signalling in neural development is the maintenance of NP self-renewal. Usp9x is capable of regulating the Notch pathway at multiple levels. Usp9x promotes Notch signalling by antagonising the degradation of Mind bomb1, as well as activating Epsin in the signal sending cell^13, 14^. Conversely, Usp9x potentially opposes Notch signalling by stabilising the Itch E3 ligase^15^, which targets the Notch intracellular domain (NICD) for proteasomal degradation in the signal receiving cell. Although no direct evidence has been reported on Usp9x regulation of Wnt signalling, the catalytic domain of Usp9x binds β-catenin and increases the half-life of the cytosolic pool^16^. Recently, two studies also identified Usp9x associated with the β-catenin destruction complex in HEK293 cells^17, 18^ suggesting another intersection between Usp9x and Wnt signalling. Therefore Usp9x interacts with both intrinsic and extrinsic mechanisms regulating NP fate specification. In the current study we sought to directly determine if Usp9x regulates NP function *in vivo* and if so, determine the molecular mechanism(s) involved.

## Results

### Usp9x regulates adherens junctions and polarity in early neural progenitors

*Nestin*-*cre* mediated deletion of *Usp9x* from mouse NPs results in their disorganisation, and that of their derivatives, within the neocortex of late stage embryos (Stegeman *et al*.^6^). However, earlier time points have not been investigated. In this study we directly examined the role of *Usp9x* in NPs using *Usp9x* conditionally deleted mice (*Usp9x* ^−/*Y*^) following mating of *Usp9x* ^*flox*/*flox*^ females with *Nestin*-*Cre* males^6^.

**Figure 1:**
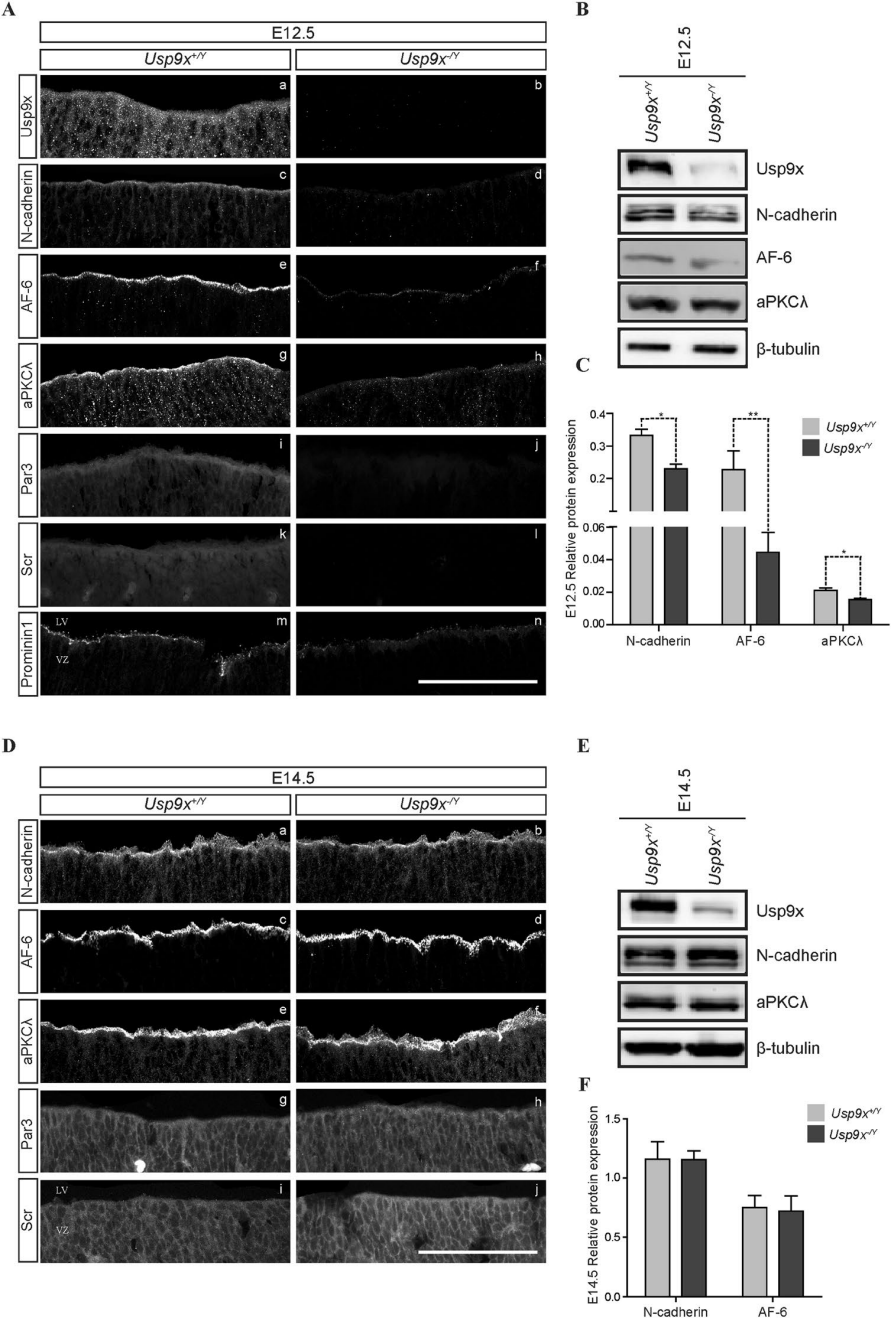
Usp9x regulates polarity and adherens junction proteins in neural progenitors. (**A**) Coronal sections of E12.5 *Usp9x* ^+/*Y*^ and *Usp9x* ^−/*Y*^ neocortices stained for Usp9x, cell adhesion and polarity markers. Usp9x protein is expressed throughout the E12.5 *Usp9x* ^+/*Y*^ ventricular zone (VZ) (a) and depleted following *Nestin*-*cre* mediated deletion (b) (each image represents n = 3 *Usp9x* ^+/*Y*^ and *Usp9x* ^−/*Y*^ embryos). Immunoreactivity of the adherens junction-associated proteins N-cadherin (c,d) and AF-6 (e,f) as well as apical polarity markers aPKCλ (g,h), Par3 (i,j), Scribble (k,l) and Prominin1 (m,n) was reduced in *Usp9x* ^−/*Y*^ NPs compared to *Usp9x* ^+/*Y*^ NPs (n = 3). (**B**) Immunoblot analyses of total Usp9x, N-cadherin, AF-6 and aPKCλ protein levels in E12.5 *Usp9x* ^+/*Y*^ and *Usp9x* ^−/*Y*^ neocortices from n = 3 embryos each. (**C**) Quantification of protein levels in (**B**) relative to β-tubulin. (**D**) Coronal sections of E14.5 *Usp9x* ^+/*Y*^ and *Usp9x* ^−/*Y*^ neocortices stained with adhesion and polarity markers (n = 3). No difference was observed between *Usp9x* ^+/*Y*^ and *Usp9x* ^−/*Y*^ neocortices for the immunostaining pattern or intensity of N-cadherin (o,p), AF-6 (q,r), aPKCλ (s,t), Par3 (u,v) or Scribble (w,x). (**E**) Immunoblot analyses of neocortical lysates confirming depletion of Usp9x and restored N-cadherin, AF-6 and aPKCλ protein levels in E14.5 *Usp9x* ^−/*Y*^ brains. (**F**) Quantification of protein levels in (**E**) relative to β-tubulin. Scale bars = 80 μm. LV- lateral ventricle, VZ- ventricular zone. All data are shown as the means ± SEM. *p < 0.05.

Usp9x protein is robustly expressed throughout the neocortex during the neurogenic period (Fig. 1A,B,E). Immunohistochemical (IHC) and immunoblot (IB) analyses of *Usp9x* ^−/*Y*^ animals compared to litter mate controls (*Usp9x* ^+/*Y*^) (n = 7) identified E12.5 as the earliest stage at which Usp9x protein was depleted from *Usp9x* ^−/*Y*^ neocortices (Fig. 1A,B). Given the disorganisation of cells in the late embryonic neocortex^6^ and established role of Usp9x in cell adhesion and polarity^3, 8, 9, 19^ we assessed the status of AJ and polarity proteins in NPs.

At E12.5, loss of Usp9x coincided with decreased levels of several AJ and polarity proteins. Immunoreactivity of N-cadherin was markedly reduced in *Usp9x* ^−/*Y*^ ventricular zones (VZs) (n = 3) (Fig. 1A, Supp. Fig. S1A). Similarly, immunoreactivity of Usp9x substrate and AJ structural protein AF-6 was also reduced (n = 3) (Fig. 1A, Supp. Fig. S1B). Significantly reduced N-cadherin and AF-6 total protein levels in *Usp9x* ^−/*Y*^ neocortices were confirmed by IB analyses (n = 3) (Fig. 1B,C). Immunoreactivity of apical polarity proteins aPKCλ, Par3, Prominin1 and the basal polarity protein Scribble was also markedly reduced in *Usp9x* ^−/*Y*^ VZs (n = 3) (Fig. 1A). Significantly reduced aPKCλ expression was confirmed by IB (n = 3) (Fig. 1B,C). Together these data suggest AJs and polarity were perturbed in E12.5 *Usp9x* ^−/*Y*^ NPs. Interestingly however, both IHC and IB analyses of the same AJ and polarity markers 48 hours later, at E14.5, failed to detect any difference between *Usp9x* ^−/*Y*^ and *Usp9x* ^+/*Y*^ NPs (Fig. 1D–F) suggesting AJs and apical-basal polarity, at least at this level of analysis, had been restored.

To further analyse the effect of Usp9x loss on adhesion and polarity, as indicated by the IHC and IB results, we conducted a microarray analysis on *Usp9x* ^−/*Y*^ E12.5 and E14.5 mouse neocortical tissues. At E12.5 over 1000 genes were differentially expressed between the *Usp9x* ^+/*Y*^ and *Usp9x* ^−/*Y*^ samples, at a significance level of p < 0.05 (n = 4 for each genotype) (Supp. Fig. S2). In concordance with perturbed polarity, the functional annotation of the differentially expressed genes at E12.5 identified cytoskeleton organisation and cell to cell signalling interactions as the most significantly affected in addition to nervous system development and function (Supp. Table S1). Despite the apparent restoration of normal NP adhesion and polarity at E14.5 (Fig. 1) nearly 1400 genes (Supp. Fig. S3) were significantly (p < 0.05) differentially expressed (n = 4) (Supp. Table S2). The affected functions included tissue morphology and, cell growth and proliferation. However, only 147 genes were common to both E14.5 and E12.5 (Supp. Table S3). Therefore, loss of Usp9x affects the expression of a large number of genes and a range of NP functions, and these differ at E12.5 and E14.5. Additionally, despite the apparent restoration of adhesion and polarity at E14.5, multiple gene networks remained perturbed in the absence of Usp9x.

### Deletion of USP9X affects the fate of apical neural progenitors

The adhesion and polarity of apical NPs in the neocortex creates a niche maintaining NP fate. Once NPs lose apical cell-cell adhesion, migration outward from the VZ occurs and their fate is restricted to either basally located intermediate progenitors or terminally differentiated neurons or glia^20, 21^. Therefore an examination of the consequences of the transient disruption of cell adhesion and polarity on the fate of *Usp9x* ^−/*Y*^ NPs was conducted.

**Figure 2:**
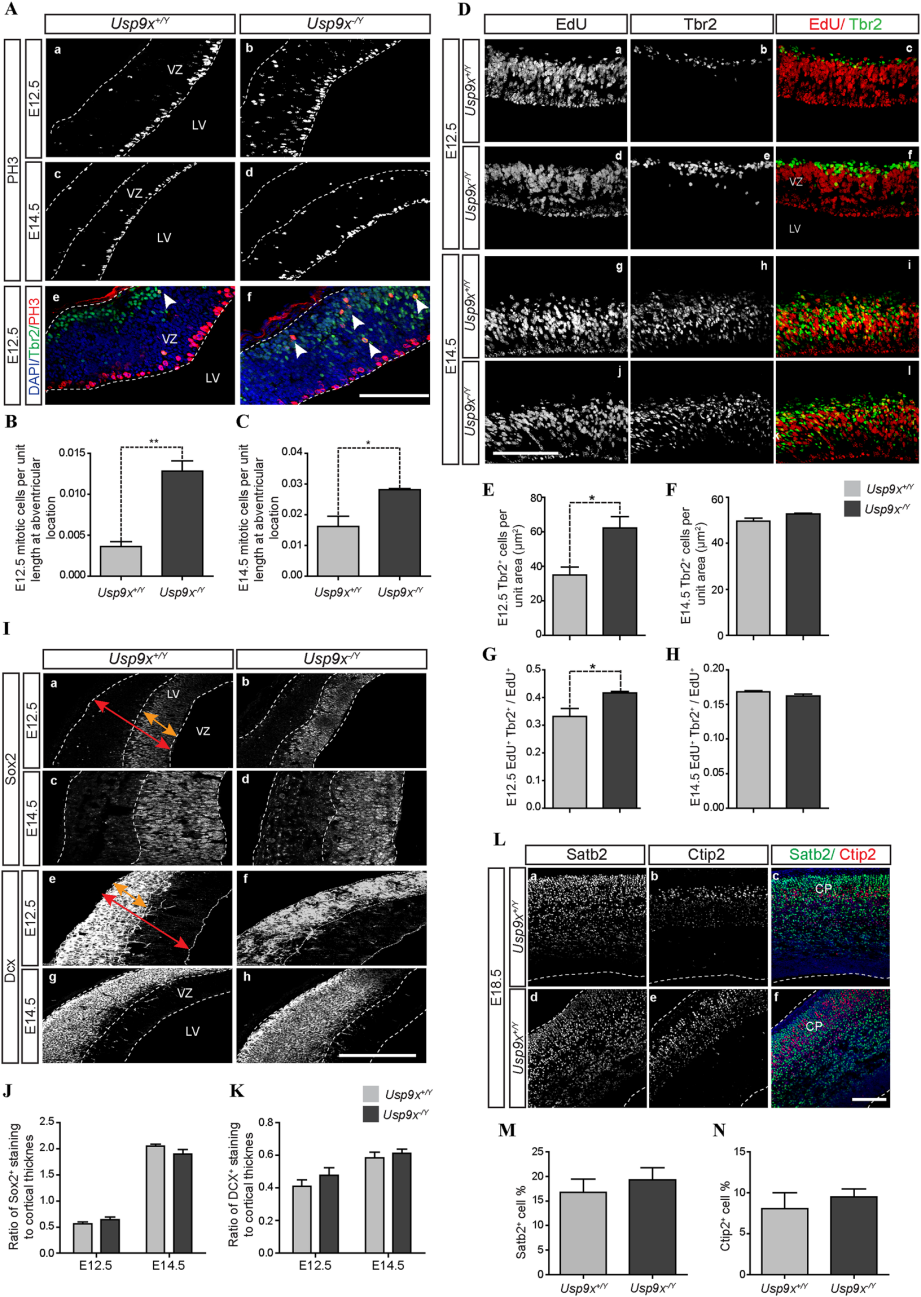
Loss of Usp9x leads to the premature appearance and ectopic location of intermediate progenitors but does not alter neurogenesis in the neocortex. (**A**) Phosphohistone3 (PH3) labels mitotic cells at the ventricular surface in E12.5 and E14.5 *Usp9x* ^+/*Y*^ neocortices (a,c), but mitotic cells were also detected in abventricular locations in *Usp9x* ^−/*Y*^ neocortices (b,d). Tbr2^+^ intermediate progenitors in *Usp9x* ^+/*Y*^ neocortex located exclusively in the subventricular zone and few labelled with PH3 (e). In *Usp9x* ^−/*Y*^ neocortex Tbr2^+^ cells in both ventricular and subventricular zones are frequently labelled with PH3 (f) (representative of n = 4 embryos for each). Number of abventricularly proliferating PH3^+^ cells was significantly increased in both E12.5 (**B**) and E14.5 (**C**) *Usp9x* ^−/*Y*^ neocortices. (**D**) Increased Tbr2^+^ intermediate progenitor cell number in *Usp9x* ^−/*Y*^ neocortices. Number of Tbr2^+^ intermediate progenitor per unit area was significantly increased in E12.5 *Usp9x* ^−/*Y*^ neocortices (**E**), but not in E14.5 *Usp9x* ^−/*Y*^ brains (**F**). Number of EdU^+^/Tbr2^+^ double-labelled cells was increased in E12.5 *Usp9x* ^−/*Y*^ neocortices (**G**), but not E14.5 *Usp9x* ^−/*Y*^ neocortices (**H**). (**I**) Loss of Usp9x does not alter overall proportions of neural progenitors or neuroblast. Coronal sections of E12.5 and E14.5 *Usp9x* ^+/*Y*^ and *Usp9x* ^−/*Y*^ neocortices stained with the neural progenitor marker Sox2 (a–d) and neuroblast marker DCX (e–h) (n = 3). Dotted lines demark the borders between the ventricular zone (VZ) and cortical plate (CP). (**J**,**K**) Comparison of relative thickness of average Sox2 and DCX staining in E12.5 and E14.5 neocortices revealed no differences between the *Usp9x* ^+/*Y*^ and *Usp9x* ^−/*Y*^ samples. (**L**) Coronal sections of E18.5 *Usp9x* ^+/*Y*^ (a–c) and *Usp9x* ^−/*Y*^ (d–f) cortices stained with deep layer neuronal marker Ctip2 (layer V) and superficial neuronal marker Satb2 (n = 3). Number of Ctip2^+^ and Satb2^+^ cells were normalised to total number of cells present as marked by DAPI staining. (**M**,**N**) No difference was observed for Ctip2^+^ and Satb2^+^ cell percentage between E18.5 *Usp9x* ^+/*Y*^ and *Usp9x* ^−/*Y*^ brains. Scale bars = 80 μm. LV- lateral ventricle, VZ- ventricular zone, CP- cortical plate. **p < 0.01; *p < 0.05.

The proliferative state of *Usp9x* ^−/*Y*^ NPs was assessed by measuring the number of NPs undergoing mitosis as marked by antibodies against phospho-histone3 (PH3). Although no difference was observed in the number of PH3-positive (PH3^+^) mitotic cells at the ventricular surface between *Usp9x* ^+/*Y*^ and *Usp9x* ^−/*Y*^ neocortices at either E12.5 or E14.5, a 6-fold increase in abventricular PH3^+^ nuclei was detected at E12.5 (*Usp9x* ^+/*Y*^ = 0.0209 ± 0.0138 PH3 + cells/μm; *Usp9x* ^−/*Y*^ = 0.1326 ± 0.1546; p < 0.01, n = 4) (Fig. 2A,B), and a 2-fold increase was evident at E14.5 (*Usp9x* ^+/*Y*^ = 0.1046 ± 0.040; *Usp9x* ^−/*Y*^ 0.1989 ± 0.038; p < 0.01, n = 4) (Fig. 2A,C) in *Usp9x* ^−/*Y*^ neocortices. To identify the nature of the abventricularly proliferating cells, sections were double-stained with the apical NP marker Pax6 and intermediate progenitor marker, Tbr2. The majority of PH3^+^ abventricular cells were also positive for Tbr2 in the *Usp9x* ^−/*Y*^ brains (n = 3) (Fig. 2A). An increased density of Tbr2^+^ cells within the neocortex in *Usp9x* ^−/*Y*^ brains at E12.5 (*Usp9x* ^+/*Y*^ = 0.33 ± 0.02 Tbr2^+^ cells/μm^2^; *Usp9x* ^−/*Y*^ = 0.41 ± 0.004 Tbr2^+^ cells/μm^2^; p < 0.05, n = 3) but not E14.5 (*Usp9x* ^+/*Y*^ = 0.16 ± 0.001 Tbr2^+^ cells/μm^2^; *Usp9x* ^−/*Y*^ 0.16 ± 0.003 Tbr2^+^ cells/μm^2^; p = 0.52, n = 3) (Fig. 2D–F) was also observed. In the *Usp9x* ^−/*Y*^ neocortex Tbr2^+^ cells were not restricted to the sub ventricular zone with many located in the ventricular zone, some of which were also PH3 positive (Fig. 2A), consistent with a premature differentiation of apical progenitors as occurs following the loss of other cell adhesion and polarity proteins^22, 23^. To investigate, Tbr2 immunostaining was conducted on brain sections following an *in vivo* 3 hour EdU pulse. In agreement with the previous results, the number of EdU^+^/Tbr2^+^ cells were significantly increased in E12.5 *Usp9x* ^−/*Y*^ brains (*Usp9x* ^+/*Y*^ = 35.00 ± 4.58; *Usp9x* ^−/*Y*^ = 62.25 ± 6.62; p < 0.05, n = 3) but not at E14.5 (*Usp9x* ^+/*Y*^ = 49.63 ± 1.37; *Usp9x* ^−/*Y*^ = 52.75 ± 0.25; p = 0.15, n = 3) (Fig. 2D,G,H). At E12.5, some EdU^+^/Tbr2^+^ cells were also located in the VZ but only in *Usp9x* ^−/*Y*^ samples (Fig. 2D).

Next the effect, if any, of increased intermediate progenitor numbers on subsequent cortical development was assessed. No difference was observed in the number of Sox2^+^ NPs in either E12.5 or E14.5 *Usp9x* ^−/*Y*^ brains (n = 3) (Fig. 2I,J). Similarly, no difference was observed for the number of Tbr1^+^ mature neurons (Supp. Fig. S4A) or Doublecortin positive (Dcx^+^) neuroblast between *Usp9x* ^+/*Y*^ and *Usp9x* ^−/*Y*^ brains in any embryonic stage (E12.5, E14.5, E16.5, E18.5) (n = 3) (Fig. 2I–K, Supp. Fig. S4B) suggesting overall neurogenesis was not overtly affected. Nevertheless, cortical lamination patterns were also assessed. Late embryonic (E18.5) brains were stained with Ctip2 and Satb2 antibodies to label deep-layered and superficial-layered neurons, respectively. No difference was observed in the number of Satb2^+^ (Fig. 2L,M) or Ctip2^+^ (Fig. 2L,N) neurons between *Usp9x* ^+/*Y*^ and *Usp9x* ^−/*Y*^ neocortices (n = 3). To ensure this was not due to the loss of early born deep-layered neurons in *Usp9x* ^−/*Y*^ brains, apoptosis was examined using cleaved Caspase3 (Cas3) antibodies and TUNEL assay at E12.5, E14.5, E16.5 and E18.5. No difference was observed in *Usp9x* ^−/*Y*^ brains with either Cas3 (Supp. Fig. S5A) or TUNEL assays (Supp. Fig. S5B) (n = 3) confirming that deep and superficial neuronal generation and/or radial migration was unaffected in *Usp9x* ^−/*Y*^ brains.

### Deletion of Usp9x increases the levels of β-catenin protein in Neural Progenitors

**Figure 3:**
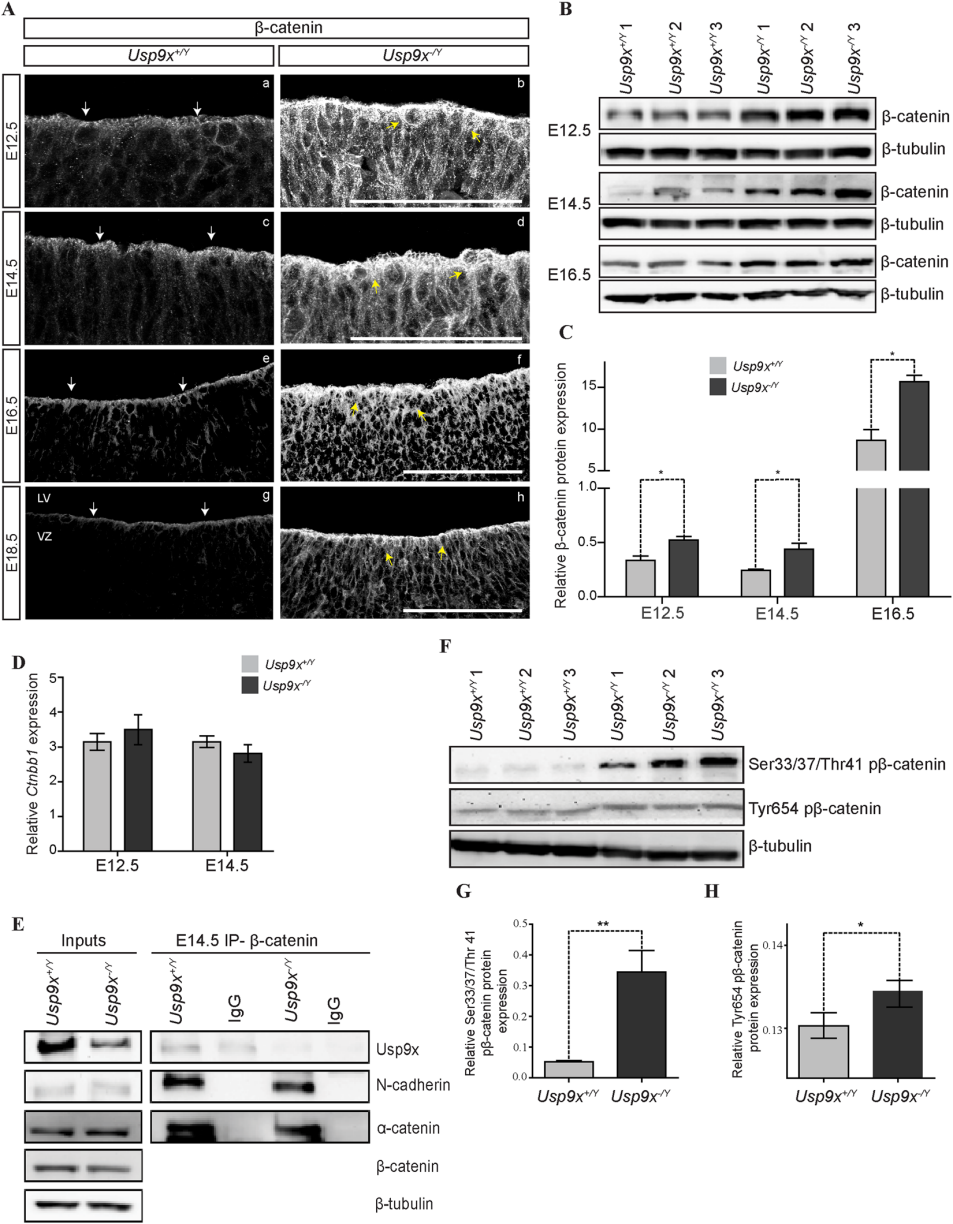
Usp9x regulates β-catenin protein levels in neural progenitors. (**A**) Coronal sections of *Usp9x* ^+/*Y*^ and *Usp9x* ^−/*Y*^ neocortices. In the *Usp9x* ^+/*Y*^ neocortex (a,c,e,g) β-catenin is predominantly localised to the apical region of the ventricular zone (VZ) (white arrows). Overall β-catenin staining was increased in *Usp9x* ^−/*Y*^ neocortices (b,d,f,h) spreading throughout the cytoplasm (yellow arrows) at all embryonic stages from E12.5 to E18.5 (n = 3 for each stage). (**B**) Immunoblot analysis confirmed increased total β-catenin levels in *Usp9x* ^−/*Y*^ cortex at each embryonic stage (n = 3). (**C**) Quantification of protein levels in (**B**) relative to β-tubulin. (**D**) Quantitative RT-PCR revealed no difference in *Ctnnb1* gene expression between *Usp9x* ^+/*Y*^ and *Usp9x* ^−/*Y*^ neocortices at E12.5 or E14.5 (n = 3). (**E**) β-catenin co-immunoprecipitated similar levels of N-cadherin and α-catenin from *Usp9x* ^+/*Y*^ and *Usp9x* ^−/*Y*^ brains. An interaction with Usp9x protein was also identified in *Usp9x* ^+/*Y*^ cortical lysate. (**F**) Immunoblot showing increased S33/37/T41 pβ-catenin and Tyr654 pβ-catenin levels in E14.5 *Usp9x* ^−/*Y*^ neorcortices (n = 3). (**G**,**H**) Quantification of protein levels in (**F**) relative to β-tubulin. Scale bars = 80 μm. LV- lateral ventricle, VZ- ventricular zone. **p < 0.01; *p < 0.05.

Surprisingly, and in contrast to other adhesion proteins, β-catenin protein levels were significantly increased in *Usp9x* ^−/*Y*^ brains in both IHC and IB analyses, across all embryonic stages examined (n = 3) (Fig. 3A–C). Even at E12.5, when other AJ and polarity proteins were downregulated, β-catenin protein was significantly increased. To determine the source of the increased β-catenin proteins, its transcriptional level was assessed by qRT-PCR analysis using RNA extracted from the neocortex at E12.5 and E14.5. No differences in β-catenin RNA levels were observed between *Usp9x* ^−/*Y*^ and *Usp9x* ^+/*Y*^ samples at either stage (n = 3) (Fig. 3D). Therefore, the increased β-catenin protein levels in *Usp9x* ^−/*Y*^ brains were due to translational or post-translational mechanisms.

β-catenin acts as both an essential linker protein in cadherin-based cell adhesion as well as a co-activator of canonical Wnt signalling^24, 25^. Under normal physiological conditions there are three cellular pools of β-catenin in NPs; (i) membrane associated β-catenin at AJs, (ii) soluble cytoplasmic β-catenin and, (iii) transcriptionally activated β-catenin in the nucleus^24^. Therefore β-catenin levels in each cellular pool were measured to identify which β-catenin species was upregulated in *Usp9x* ^−/*Y*^ NPs.

First, the cadherin associated β-catenin pool was examined by co-immunoprecipitation. The normal stoichiometry between β-catenin and N-cadherin and α-catenin was evident in E14.5 (Fig. 3E) and E12.5 (Supp. Fig. S6A) *Usp9x* ^−/*Y*^ brains suggesting the increased β-catenin protein levels were not attributable to its accumulation at AJs. The cytoplasmic and nuclear pools of β-catenin were also assessed. Under physiological conditions the majority of cytoplasmic β-catenin is efficiently bound, phosphorylated and ubiquitylated by the β-catenin destruction complex^17^. This sub-population of β-catenin can be identified by the phosphorylation of the amino acid residues Serine 33/37 and Threonine 41 (pβ-catenin33/37/41). Strikingly, a 6-fold increase in pβ-catenin33/37/41 was detected in *Usp9x* ^−/*Y*^ brains at E14.5 (n = 3) (Fig. 3F,G). In addition a slight, but significant, increase in the transcriptionally activated form of β-catenin (Tyr654 pβ-catenin)^26^ was also detected in *Usp9x* ^−/*Y*^ lysates at E14.5 (n = 3) (Fig. 3F,H).

### Absence of Usp9x alters relative ratios of proteins in the β-catenin destruction complex

A direct physical interaction between Usp9x and β-catenin has been observed in several *in*-*vitro* systems^9, 16, 27^. In line with these studies, Usp9x was co-immunopercipitated with β-catenin from E14.5 *Usp9x* ^+/*Y*^ neocortical lysates extending this interaction *in*-*vivo* (Fig. 3E). However, in contrast to previous studies, which indicated Usp9x positively regulates β-catenin protein levels, significantly increased pβ-catenin33/37/41 and Tyr654 pβ-catenin levels observed in *Usp9x* ^−/*Y*^ neocortices implied a novel regulatory mechanism in NPs.

**Figure 4:**
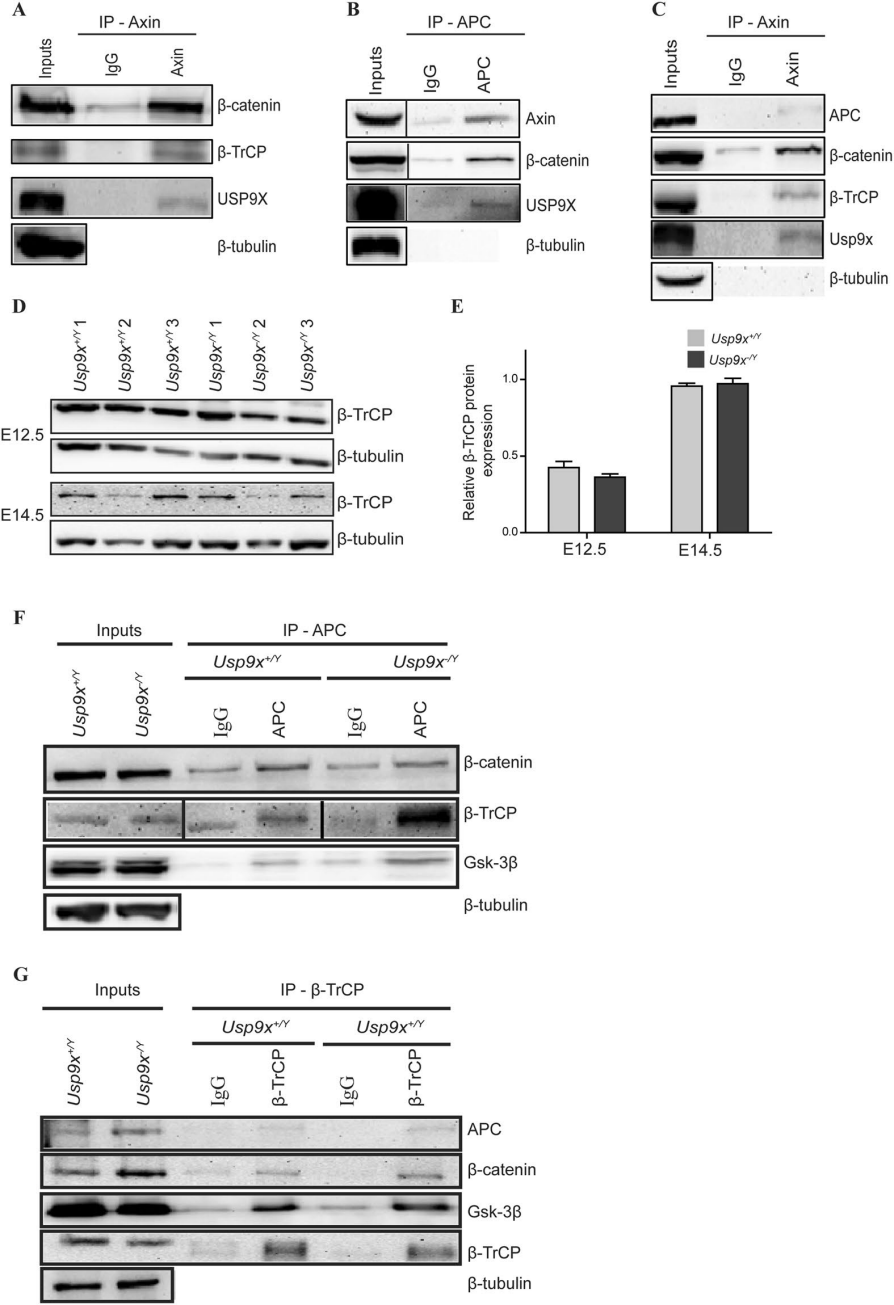
Relative ratios of β-catenin destruction complex components are altered in *Usp9x* ^−/*Y*^ neocortices. (**A**–**C**) Usp9x is part of the β-catenin destruction complex. Endogenous USP9X co-immunoprecipitated from HEK293 lysate along with components of the β-catenin destruction complex using Axin antibody (**A**) and APC antibody (**B**). (**C**) Usp9x immunoprecipitated by Axin antibody from E14.5 *Usp9x* ^+/*Y*^ cortical lysate along with other components of the destruction complex. (**D**) Immunoblot analysis of β-TrCP protein levels in *Usp9x* ^+/*Y*^ and *Usp9x* ^−/*Y*^ neocortices at E12.5 and E14.5 (n = 3). (**E**) Quantitation of (**D**) revealed no difference in total β-TrCP protein levels between *Usp9x* ^+/*Y*^ and *Usp9x* ^−/*Y*^ neocortices at E12.5 or E14.5. (**F**) Components of destruction complex were co-immunoprecipitated by anti-APC antibody from E14.5 *Usp9x* ^+/*Y*^ and *Usp9x* ^−/*Y*^ cortical lysate. Relatively more β-TrCP and GSK-3β were immunoprecipitated from *Usp9x* ^−/*Y*^ compared to *Usp9x* ^+/*Y*^. (**G**) Co-immunoprecipitation of destruction complex components using anti-β-TrCP antibody. Increased GSK-3β protein level co-immunoprecipitated from *Usp9x* ^−/*Y*^ cortical lysate confirming increased interaction with β-TrCP.

Interestingly, two studies using mass spectrometry analysis identified Usp9x as a component of the β-catenin destruction complex in HEK293 cells^17, 18^. First, we confirmed these results by co-immunoprecipitating Usp9x with Axin and APC from HEK293 cell lysates, using only endogenous proteins in each case (Fig. 4A,B). Using the same conditions endogenous Axin co-immunoprecipitated with Usp9x, other components of the destruction complex, APC and GSK3β, as well as the substrate β-catenin, from E14.5 neocortical lysates, which contain a high percentage of NPs (Fig. 4C). These data confirm that a subset of Usp9x associates with the β-catenin destruction complex in HEK293 cells and NPs *in*-*vivo*.

The increased levels of pβ-catenin33/37/41 in the *Usp9x* ^−/*Y*^ neocortex suggested decreased proteasomal degradation of β-catenin, which is targeted for ubiquitylation by the E3 ligase β-TrCP. However, no difference in total β-TrCP protein level was observed by WB at either E12.5 or E14.5 (n = 3) (Fig. 4D,E). Ubiquitylation of pβ-catenin33/37/41 occurs within the destruction complex^17^. These results raised the prospect that loss of Usp9x perturbed the assembly, composition and/or activity of the destruction complex. No difference was observed in total APC or GKS-3β protein levels in *Usp9x* ^−/*Y*^ brains at E12.5 or E14.5 (n = 3) (Supp. Fig. S6B–I). Next the assembly of the destruction complex in *Usp9x* ^−/*Y*^ brains was assessed at E14.5, the earliest time point with increased cytoplasmic β-catenin but normal levels of other adhesion and polarity proteins. The destruction complex is a static multiprotein complex therefore its protein composition remains constant and so immunoprecipitation of endogenous proteins should reveal structural differences between *Usp9x* ^−/*Y*^ and *Usp9x* ^+/*Y*^ samples^17, 28^. All destruction complex components analysed co-immunoprecipitated from *Usp9x* ^−/*Y*^ brains (Fig. 4F, representative of two biological replicates), suggesting the absence of Usp9x did not prevent its assembly. Indeed, unexpectedly higher levels of Gsk-3β and β-TrCP co-immunoprecipitated from *Usp9x* ^−/*Y*^ cortical lysates (Fig. 4F) implying their increased interaction/presence within the complex. Co-immunoprecipitation using β-TrCP antibody corroborated the APC result revealing increased levels of GKS-3β protein from *Usp9x* ^−/*Y*^ lysates (Fig. 4G).

### Usp9x antagonises Wnt signalling in neural progenitors

**Figure 5:**
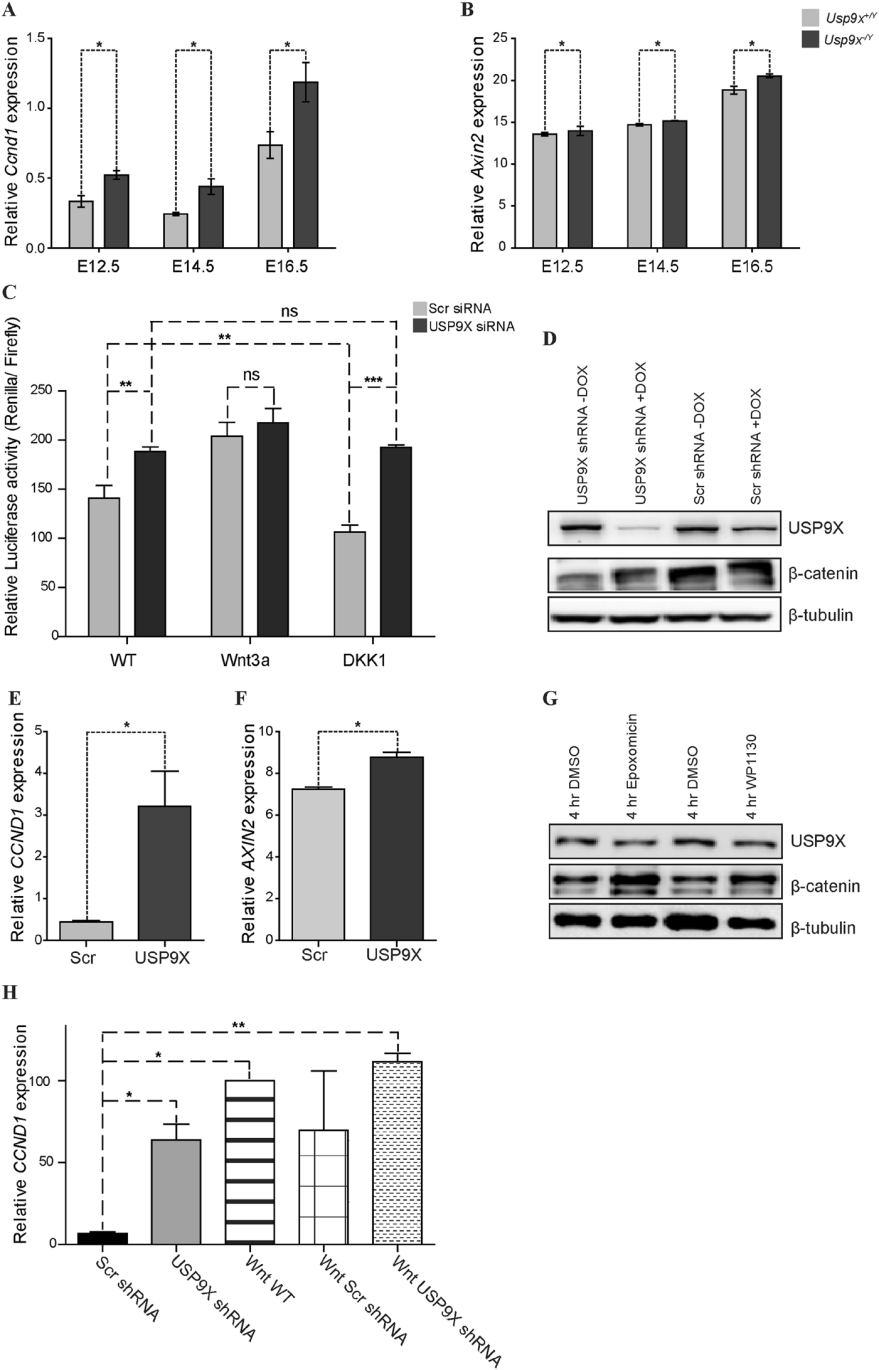
Depletion of Usp9x increases Wnt target gene expression. (**A**,**B**) qRT-PCR analysis of RNA isolated from E12.5, E14.5 and E16.5 neocortices of *Usp9x* ^+/*Y*^ and *Usp9x* ^−/*Y*^ (n = 3). Canonical Wnt signalling target genes *Ccnd1* (**A**) and *Axin2* (**B**) were up regulated in the *Usp9x* ^−/*Y*^ neocortices compared to *Usp9x* ^+/*Y*^ in all tested embryonic stages. (**C**) TCF-TOPFlash reporter activity was significantly increased in USP9X siRNA transfected HEK293 compared to scrambled nonsense siRNA transfected cells. Addition of Wnt3a increased TOPFlash reporter activity in scrambled siRNA cells to a level similar to Usp9x siRNA treated cells (n = 3). Addition of the Wnt antagonist DKK1, significantly reduced TOPFlash reporter activity only in the Scramble siRNA HEK293 cells (n = 3). (**D**) Increased total β-catenin protein levels in USP9X-depleted ReNcell VM. (**E**,**F**) Expression of canonical Wnt signaling target genes, *CCND1* and A*XIN2* was significantly increased in USP9X-depleted ReNcell VM cells. (**G**) Inhibition of USP9X deubiquitylating activity in ReNcell VM cells by WP1130 increased total β-catenin levels to a level similar to the proteasome inhibitor epoxomicin. DMSO was used as the vehicle control. (**H**) Quantitative RT-PCR analysis of *CCND1* gene expression identified significant increases in USP9X-depleted ReNcell VMs compared with those expose to Scrambled shRNA. Treatment with Wnt3a increased *CCND1* expression in untreated (WntWT) as well as scrambled and Usp9x shRNA treated ReNcell VMs. Statistical significance was assessed by one-way ANOVA, followed by Tukey’s post-test. ***p < 0.001; **p < 0.01; *p < 0.05.

Next we examined whether the increased β-catenin protein levels, especially Tyr654 pβ-catenin, in the *Usp9x* ^−/*Y*^ brains activated the Wnt signalling pathway by assaying target gene levels using qRT-PCR. Consistent with the preceding results significantly increased *Ccnd1* and *Axin2* gene expression levels were observed in the *Usp9x* ^−/*Y*^ brains at E12.5, E14.5 and E16.5 (n = 3) (Fig. 5A,B). However, the levels of target gene induction varied widely (50% to 80% for *Ccnd1*; 5% for *Axin2*). Ingenuity pathway analysis conducted on the microarray data independently identified Wnt signalling as one the most significantly affected pathways in *Usp9x* ^−/*Y*^ brains (Table S3). Further, qRT-PCR analysis confirmed changes in the expression level of the Wnt pathway genes identified by Ingenuity, in E12.5 *Usp9x* ^−/*Y*^ brains (Supp. Fig. S7) but only *Sox4* and *Sox1* were significantly affected at E14.5. Although these genes impact upon Wnt signalling they are not direct Wnt targets.

Therefore, to test if loss of Usp9x can directly activate Wnt signalling, TCF-TOPflash reporter assays were performed on HEK293 cells from which USP9X was depleted with siRNA. USP9X protein level was depleted 72 hours after siRNA transfection and a clear increase in pβ-catenin33/37/41 protein level was observed (Supp. Fig. S6J). Increased luciferase activity was also observed in USP9X-depleted HEK293 cells (n = 3) (Fig. 5C). The addition of optimised concentrations of exogenous Wnt3a increased luciferase activity in HEK293 cells treated with control shRNA. However, exogenous Wnt3a did not induce higher levels of luciferase activity than depletion of USP9X alone (Fig. 5C). In contrast, addition of the extracellular Wnt inhibitor DKK1, did not alter luciferase activity in USP9X-depleted cells but significantly reduced it in control shRNA treated cells (n = 3) (Fig. 5C). Therefore depletion of USP9X is capable of directly activating Wnt target gene expression.

The embryonic neocortex is composed of multiple types of NPs and fate restricted neural cells. Therefore, to test the effect of Usp9x loss on Wnt target gene expression in a homogeneous NP population, USP9X was depleted in the human NP cell line, ReNcell VM, using doxycycline inducible shRNA against *USP9X* ^29^. 72 hours after shRNA induction, USP9X protein was almost completely depleted in these cells (Fig. 5D). Consistent with the *in*-*vivo* results, USP9X-depleted ReNcell VM cells showed increased β-catenin protein levels (Fig. 5D), a 10-fold increase in *CCND1* gene expression, and a modest (25%) but significant increase in *AXIN2* expression (n = 3) (Fig. 5E). To determine whether the USP9X DUB activity is required for β-catenin regulation, ReNcell VM cells were treated with the DUB inhibitor WP1130, which inhibits the USP9X DUB activity without affecting its protein levels^30–32^. Similar to *in*-*vivo* and shRNA-induced USP9X depletion, β-catenin protein level was markedly increased in WP1130 treated ReNcell VM cells to the same level as proteasome inhibition (epoxomicin treatment) (Fig. 5G). A caveat to these results is that WP1130 inhibition is not exclusive to USP9X^30^. However as all other DUBs stabilise β-catenin protein levels^33–36^ their WP1130 inhibition would result in decreased β-catenin levels, the opposite of that observed. Finally, as in HEK293 cells, depletion of USP9X increased *CCND1* gene expression to levels approaching that of exogenous Wnt3a (Fig. 5H) in ReNcell VM NPs. The depletion of USP9X in the presence of Wnt3a did not further increase *CCDN1* expression. Therefore USP9X depletion increased *CCND1* expression in ReNcell VM NPs and TOPFlash induction in HEK293 cells but the effect on *CCND1* was greater.

### Notch signalling is activated in Usp9x^−/*Y*^ brains

Over-expression of stabilised forms of β-catenin and activation of Wnt signalling induce both NP self-renewal and neurogenesis during early and mid-neurogenic periods, respectively^37–40^. However, no overt changes in either neurogenesis or total NP numbers were observed in early (Fig. 2) or late (Stegeman *et al*.^6^) *Usp9x* ^−/*Y*^ brains. One possible explanation for this apparent discrepancy could reside with Usp9x’s regulation of other signalling pathways including Notch, which opposes Wnt signalling in multiple progenitor populations^41–43^. Usp9x regulates components of the Notch pathway in both signal sending and receiving cells^44^. Therefore, we hypothesised that Notch signalling might also be altered in *Usp9x* ^−/*Y*^ NPs.

**Figure 6:**
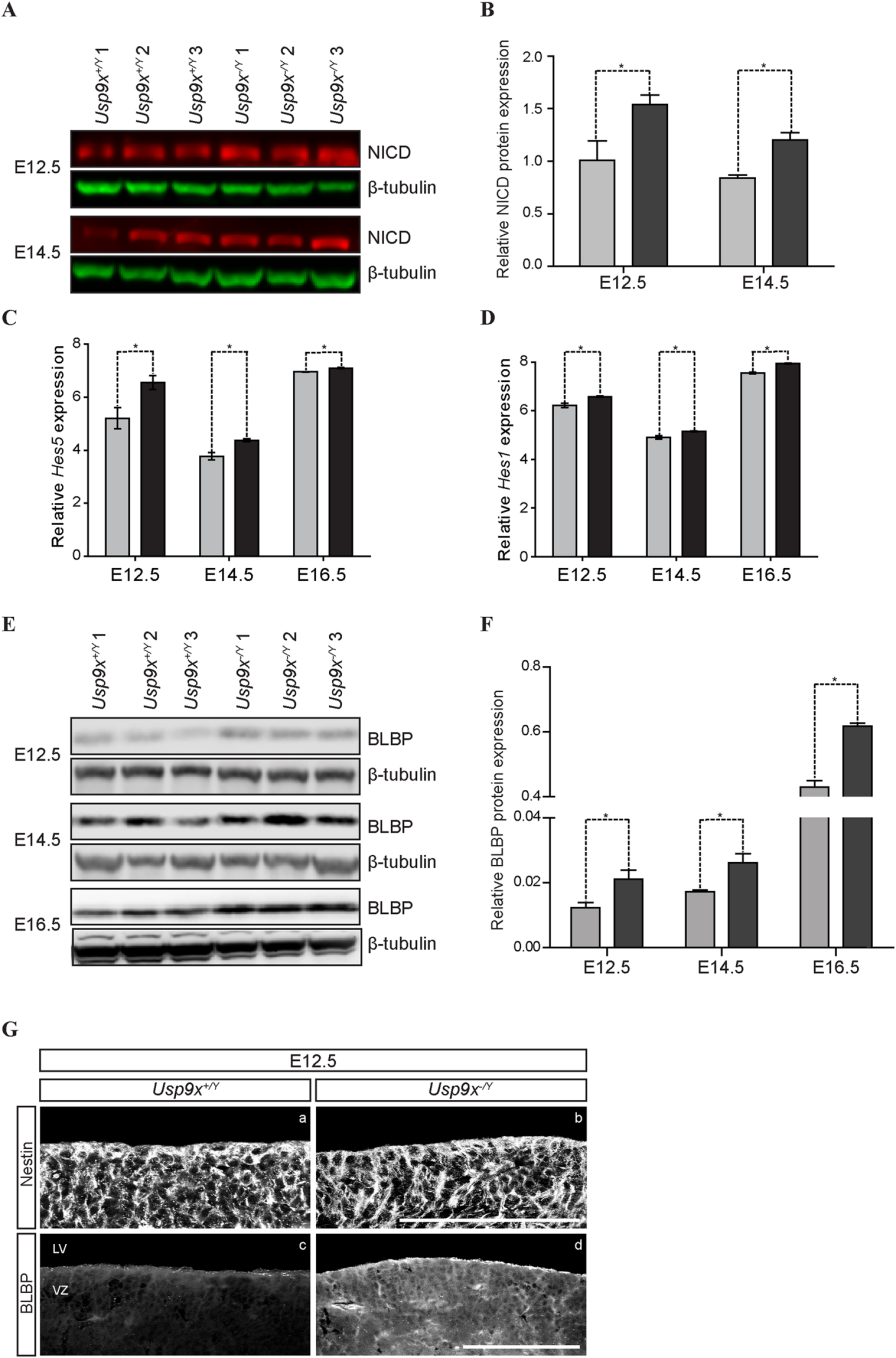
Increased levels of Notch signalling pathway components in *Usp9x* ^−/*Y*^ neocortices. (**A**) Immunoblot analysis showing increased levels of Notch intracellular domain (NICD) in E12.5 and E14.5 *Usp9x* ^−/*Y*^ neocortices (n = 3). (**B**) Quantification of relative NICD protein levels in (**A**) after normalisation to β-tubulin. (**C**) qRT-PCR analyses showing slightly increased *Hes1* and *Hes5* gene expression in E12.5, E14.5 and E16.5 *Usp9x* ^−/*Y*^ neocortices (n = 3). (**E**) Immunoblot analysis showing increased BLBP protein levels in E12.5, E14.5 and E16.5 *Usp9x* ^−/*Y*^ neocortices (n = 3). (**F**) Quantification of relative BLPB protein levels in (**E**) after normalisation to β-tubulin. (**G**) Coronal sections of E12.5 *Usp9x* ^+/*Y*^ and *Usp9x* ^−/*Y*^ neocortices stained with Nestin and BLBP (n = 3). BLBP displayed higher immunoreactivity in *Usp9x* ^−/*Y*^ neocortices (c,d) No difference was observed for Nestin expression (a,b). Scale bars = 80 μm. LV- lateral ventricle, VZ- ventricular zone. *p < 0.05.

To this end, NICD levels were assessed in *Usp9x* ^−/*Y*^ brains and a 50% increase was detected at both E12.5 and E14.5 (p < 0.05) (Fig. 6A,B) raising the possibility of increased Notch signalling. qRT-PCR analysis of Notch target genes in E12.5, E14.5 and E16.5 revealed *Hes5* expression was increased in *Usp9x* ^−/*Y*^ cortex (20% at E12.5; 16% at E14.5; p < 0.05) (Fig. 6C). Much smaller increases were detected for *Hes1* at E12.5 and 14.5 and both *Hes5* and *Hes1* at E16.5 (Fig. 6C,D). However, *Hes5* more accurately reflects Notch activity than *Hes1* in NPs^45, 46^ suggesting Usp9x depletion resulted in Notch activation. To examine whether the increased NICD level induced a physiological response, brain lipid binding protein (BLBP) expression, a NP-specific Notch target^47^ was examined in the *Usp9x* ^−/*Y*^ NPs. Consistent with preceding results increased BLBP protein expression was observed in E12.5, E14.5 and E16.5 *Usp9x* ^−/*Y*^ neocortical tissue (n = 3) (Fig. 6E,F). Increased BLBP expression may also have resulted from cortical hypertrophy or premature gliogenesis. However, no difference was observed in Nestin or GFAP expression in *Usp9x* ^−/*Y*^ cortices at E12.5 (n = 3) (Fig. 6G) or E18.5 (n = 3) (Supp. Fig. S8A,B), respectively, suggesting increased BLBP does not reflect changes in cell populations. Similarly, increased *HES5* and *HES1* expression were observed in the USP9X-depleted ReNcell VM NPs as well (Supp. Fig. S8C,D).

### Numb protein expression is regulated by Usp9x

**Figure 7:**
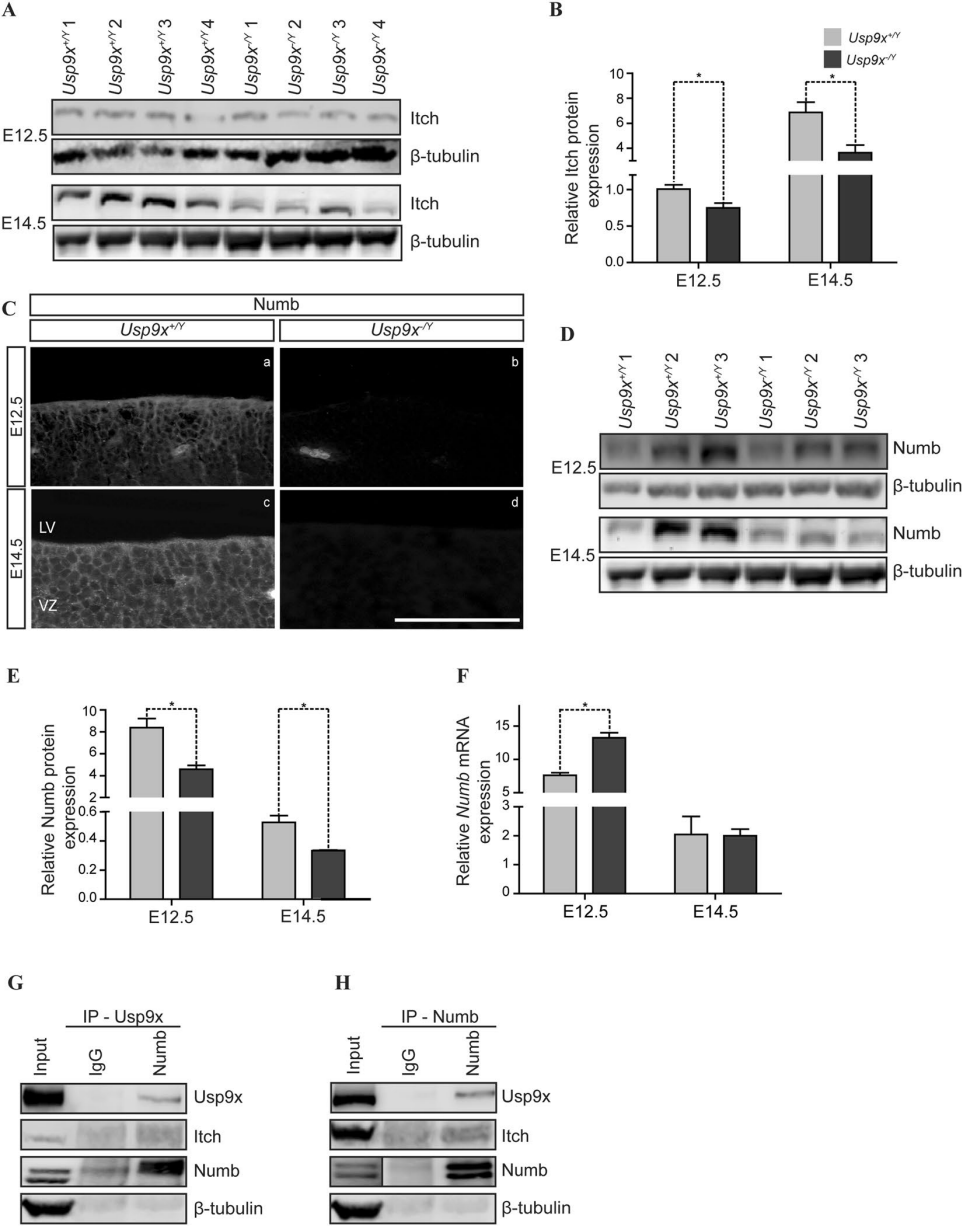
Numb is a novel binding partner of Usp9x. (**A**) Immunoblot analysis showing reduced Itch protein levels in E12.5 and E14.5 *Usp9x* ^−/*Y*^ neocortices (n = 4). (**B**) Quantification of Itch protein levels in (**A**) after normalisation to β-tubulin. (**C**) Coronal sections of E12.5 and E14.5 *Usp9x* ^+/*Y*^ and *Usp9x* ^−/*Y*^ neocortices stained with Numb (n = 3), showing reduced immunoreactivity in *Usp9x* ^−/*Y*^ neocortices. (**D**) Immunoblot analysis of reduced Numb protein levels in E12.5 and E14.5 *Usp9x* ^−/*Y*^ brains (n = 3). (**E**) Quantification of relative Numb protein levels in (**D**) after normalisation to β-tubulin. (**F**) Quantification of *Numb* mRNA level, relative to GAPDH, by qRT-PCR at E12.5 and E14.5. n = 4 for each genotype at each stage. (**G**) Co-immunoprecipitation analysis conducted on E14.5 cortical lysates using Usp9x antibody, immunoprecipitated both Numb and Itch proteins. (**H**) Co-immunoprecipitation conducted on E14.5 cortical lysates using Numb antibodies immunoprecipitated Usp9x and Itch proteins confirming these proteins forms a complex. Scale bars = 80 μm. LV- lateral ventricle, VZ- ventricular zone. *p < 0.05.

Increased NICD levels in *Usp9x* ^−/*Y*^ brains could result from increased receptor activation and/or impaired NICD degradation. Mind bomb1 is an Usp9x substrate, which promotes Notch signalling by activating ligands^13, 48^ in turn leading to Notch receptor activation and increased NICD levels. However, no differences were observed in Mind bomb1 expression in E12.5 or E14.5 *Usp9x* ^−/*Y*^ brains (n = 3) (Supp. Fig. S9). Next, the Usp9x substrate E3 ligase Itch was examined. Itch expression was significantly reduced in E12.5 and E14.5 *Usp9x* ^−/*Y*^ brains (n = 4) (Fig. 7A,B) implying increased NICD expression may, at least partially, be due to decreased Itch targeting of NICD for proteasomal degradation. In signal receiving cells, Numb initiates the binding of Itch to membrane tethered NICD^49^ and is a well-established Notch antagonist in NPs^50^. Numb protein expression was also significantly reduced in the *Usp9x* ^−/*Y*^ neocortex at E12.5 and E14.5 as determined by both IHC and IB analyses (n = 3) (Fig. 7C–E). Interestingly, qRT-PCR conducted on RNA extracted from E12.5 cortices showed increased *Numb* expression in *Usp9x* ^−/*Y*^ brains, however no difference was observed in E14.5 *Usp9x* ^−/*Y*^ brains (Fig. 7F). These data indicate that the reduced Numb protein expression in *Usp9x* ^−/*Y*^ brains is not due to decreased transcription.

**Figure 8:**
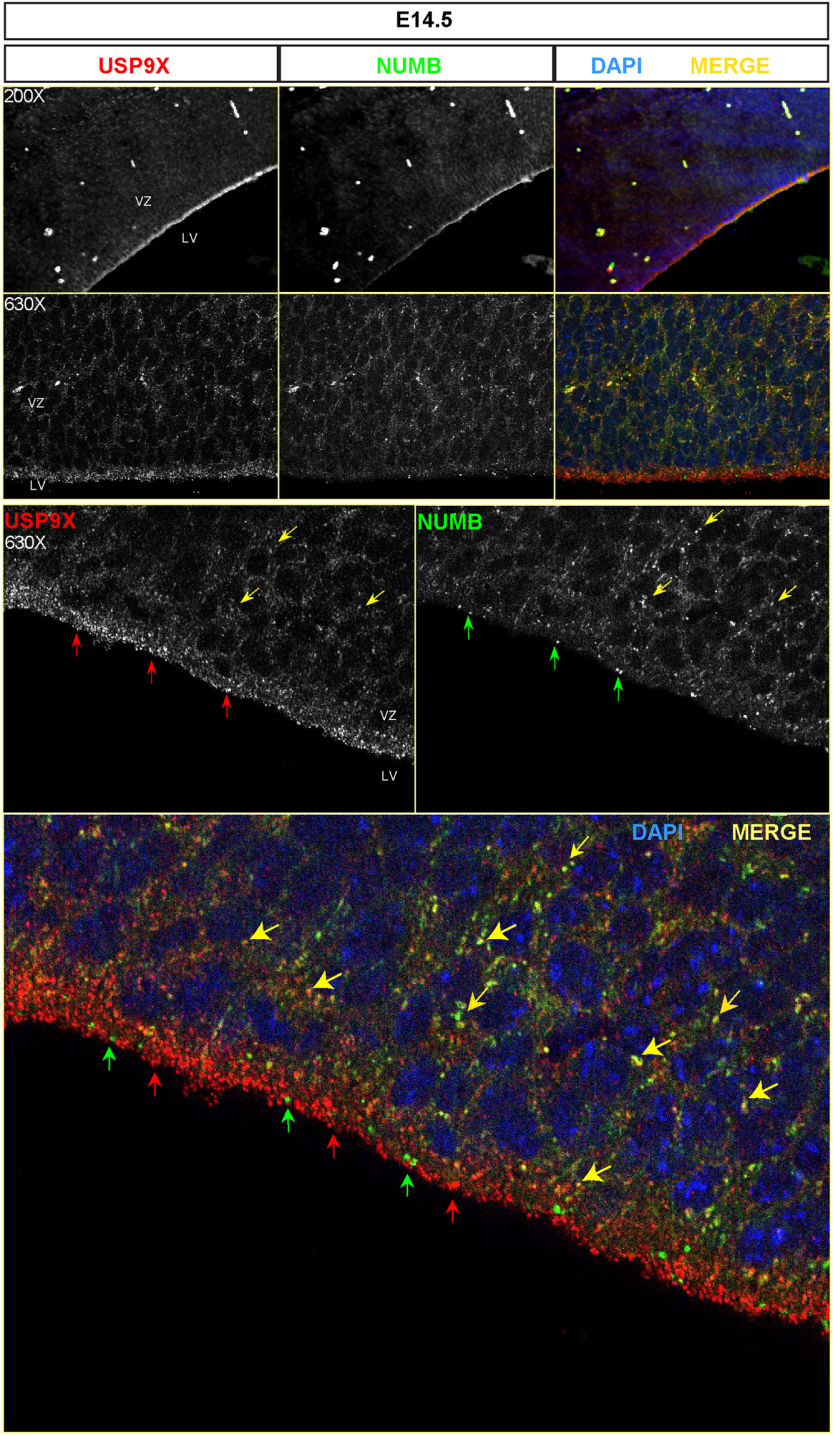
Usp9x co-localises with Numb in embryonic mouse brains. Coronal sections of E14.5 neocortices immunostained with Usp9x (red) and Numb (green) antibodies. Yellow arrows show extensive co-localization of Usp9x and Numb in the basal domain of NPs in the VZ. However Usp9x (red arrows) and Numb (green arrows) do not colocalise in the apical region above the nuclei (DAPI, blue) closest to the ventricle. LV- lateral ventricle, VZ-ventricular zone.

The above results imply a direct correlation between Usp9x and Numb protein levels in neural cells. Numb is ubiquitylated by two RING E3 ligases Murine double minute (MDM2)^51^ and LNX^52^ targeting it for proteasomal degradation. However, no DUB has been reported to interact with NUMB. Therefore we sought to determine whether Usp9x binds Numb in the embryonic neocortex. Co-immunoprecipitation with Usp9x antibody from E14.5 *Usp9x* ^+/*Y*^ cortical lysates detected a strong interaction with Numb and, to a lesser extent, Itch (Fig. 7G). Reciprocal co-immunoprecipitation with Numb antibody immunoprecipitated Usp9x and Itch (Fig. 7H). In addition IHC analysis revealed extensive co-localisation of Usp9x and Numb in puncta in the basal, but not apical, domain of NPs in the VZ in E14.5 brains (n = 3) (Fig. 8). These results suggest that Usp9x positively regulates Numb in mouse NPs and that diminished Numb and Itch levels results in increased Notch signalling.

## Discussion

NP fate is regulated by both extrinsic signalling pathways as well as intrinsic factors such as cell-cell adhesion and apical-basal cell polarity^21^. Usp9x is required for normal brain development^6^ but it was not clear what role, if any, it played in NPs. *Nestin*-*Cre* expression resulted in the complete deletion of Usp9x protein from NPs by E12.5 and expression of both AJ and polarity proteins was significantly decreased in *Usp9x* ^−/*Y*^ NPs at this stage (Fig. 1A–C). Functional annotation of microarray analysis identified cellular assembly, organisation, morphology and development as the most significantly affected pathways in E12.5 *Usp9x* ^−/*Y*^ brains (Table S1). However, 48 hours later, at E14.5, all AJ and polarity proteins were detected at normal levels and localisations in *Usp9x* ^−/*Y*^ NPs (Fig. 1D–F).

One interpretation of these data is that Usp9x regulates the establishment, rather than maintenance, of NP adhesion/polarity similar to its role in other polarised epithelia. In cultured MDCKII cells Usp9x facilitates *de novo* tight junction assembly by regulating the temporal and spatial expression of the tight junction protein EFA6^8^. Loss of Usp9x did not affect the morphology or function of mature tight junctions in MDCKII cells, but it significantly delayed their *de novo* formation after a calcium switch^8^. Similarly in T84 colon epithelial cells Usp9x interacted and colocalized with the cadherin-catenin heterodimer at points of protein trafficking in semi confluent cells establishing cell adhesion, but no association was detected with fully mature AJ complexes^9^. In NPs *in*-*vivo* Usp9x co-immunoprecipitated with both N-cadherin and β-catenin and the N-cadherin/β-catenin heterodimer was intact in E12.5 *Usp9x* ^−/*Y*^ brains (Supp. Fig. S6A), despite N-cadherin protein being significantly reduced at the apical domain. Therefore Usp9x may regulate trafficking of the N-cadherin/β-catenin heterodimer in NPs during AJ establishment as suggested in other epithelia. AJs and apical-basal cell polarity are functionally and structurally interconnected in NPs such that disrupting one perturbs the other^53, 54^. Therefore, delayed AJs establishment in *Usp9x* ^−/*Y*^ NPs would likely have been associated with a transient apical-basal polarity disruption in E12.5 *Usp9x* ^−/*Y*^ NPs. Similar conclusions have been reached following studies of MAL-3 knockout NPs, in which MAL-3 was shown to be important for the establishment rather than maintenance of AJs and polarity^55^. Likewise, the importance of Cdc42 in AJ establishment^22^ and in contrast, importance of RhoA in AJ maintenance^20^ was concluded based on stage-specific cortical tissue dysmorphia in Cdc42 and RhoA mutant mice. Regarding the transient nature of the AJ and polarity disruption in *Usp9x* ^−/*Y*^ NPs, it remains possible that another DUB may compensate for the loss of Usp9x however the microarray analysis failed to detect up regulation of other DUBs in *Usp9x* ^−/*Y*^ brains.

In developing mammalian brains AJs contribute to the stem niche by connecting adjacent NPs, thus facilitating cell-cell communications that promote self-renewal. Disrupting AJs results in the premature differentiation of apical NPs into more fate restricted cell types^21^. Loss of Usp9x resulted in the increased number and density of Tbr2^+^ intermediate progenitors throughout the cortex, including many ectopically located in the VZ in E12.5 *Usp9x* ^−/*Y*^ neocortex (Fig. 2A–F). Some Tbr2^+^ cells in the VZ also labelled with EdU or PH3 consistent with the premature differentiation of apical NPs to intermediate progenitors as occurs following the loss of other adhesion and polarity proteins including the small GTPases Cdc42^22^ and Rac1^23^ as well as N-cadherin^56^, β-catenin^57^ and RhoA^20^. However, whilst *Usp9x* deletion induced the premature appearance of intermediate progenitors, it did not alter the overall production of neurons, as no difference was observed in the total neurogenesis or lamination pattern in *Usp9x* ^−/*Y*^ brains (Fig. 2G–L). Similarly, *Nestin*-*Cre* mediated deletion of aPKCλ also perturbed AJ formation and increased abventricular proliferation, but did not affect overall neurogenesis^54^. No difference was observed in either cleaved Caspase3 or TUNNEL assays (Supp. Fig. S6A,B) suggesting cell death is unlikely to balance any persistent increase in neuronal numbers in the absence of Usp9x. Therefore the data presented herein strongly supports a role for Usp9x in the regulation of NP adhesion and polarity during neocortical development.

In stark contrast to other AJ and polarity proteins, a clear increase in β-catenin protein was detected in *Usp9x* ^−/*Y*^ neocortices at all embryonic stages from E12.5 onward suggesting Usp9x regulates β-catenin independently of its AJs function. As no change was observed in β-catenin mRNA levels, a translational and/or posttranslational defect was proposed to drive the β-catenin protein up regulation in *Usp9x* ^−/*Y*^ brains.

Significantly increased pβ-catenin33/37/41 levels in *Usp9x* ^−/*Y*^ brains suggested two things, (i) that the components required to progressively phosphorylate β-catenin are present and functional in the destruction complex and, (ii) that either the progression of phosphorylated β-catenin or the activity of the ubiquitylation machinery within the destruction complex is hindered by the absence of Usp9x. Immunoblot analysis revealed the cellular levels of destruction complex proteins were unaltered and, immunoprecipitation confirmed the endogenous proteins interacted in the absence of Usp9x (Figs 4D, S4B–I). However, the increased association between β-TrCP and GSK-3β in *Usp9x* ^−/*Y*^ brains (Fig. 4F,G) imply the stoichiometry of the complex may be altered. Normally pβ-catenin33/37/T41 is ubiquitylated within the destruction complex and rapidly degraded by the proteasome^17^ as evident by minimal levels of this form of β-catenin in wild type neocortices (Fig. 3F). That WP1130 treatment of ReNcell VM produced an equivalent increase in β-catenin levels as proteasome inhibition strongly indicates the importance of deubiquitylase activity for this regulatory step. The precise mechanism whereby Usp9x regulates ubiquitylation within the destruction complex is yet to be elucidated as little is known about the sequential biochemical reactions involved. However, a recent study demonstrated that during the Wnt-on state, β-TrCP monoubiquitylates Gsk-3β^58^. This did not result in GSK-3β degradation nor affect its enzymatic activity, but instead increased the association between GSK-3β and β-TrCP which suppressed β-catenin recruitment to β-TrCP, leading to long-term inhibition of β-catenin ubiquitylation^58^. The increased interaction between GSK-3β and β-TrCP identified by both direct co-immunoprecipitation or indirectly via APC in Usp9x^−/*Y*^ cortices (Fig. 4F,G) suggests β-TrCP may not dissociate from GSK-3β in the absence of Usp9x. Others have reported similarly higher levels of S33/37/T41 pβ-catenin in the cancer cell line SW480, which carries a truncated form of APC. However, similar to Usp9x^−/*Y*^ brains both β-TrCP and Gsk-3β remained within the destruction complex^59^.

β-catenin protein levels are regulated by several DUBs including Usp7, Usp34, Usp4 and Usp47^33–36^, which oppose its proteasomal degradation. In contrast to the data presented here others have reported a positive correlation between Usp9x and β-catenin protein levels. Previously Taya, *et al*.^16^ reported that over expression of Usp9x’s catalytic domain rescued β-catenin from proteasomal degradation in L cells. This apparently contradictory result may be due to either, (i) the differing roles of Usp9x in L-cells and embryonic NPs and/or (ii) the overexpression of Usp9x’s catalytic domain alone raises concerns about substrate specificity. USP9X also interacts with the cadherin-catenin homodimer at points of protein trafficking in semi-confluent T84 epithelial cells implying a subpopulation of USP9X interacts with the AJ-bound β-catenin (Murray *et al*.^9^). The co-immunoprecipitation of Usp9x with N-cadherin and β-catenin in the embryonic neocortex suggests this interaction also occurs in NPs *in vivo*. However this pool of β-catenin is not affected following Usp9x deletion and so did not alter total β-catenin levels.

The increase β-catenin levels raised the possibility that Wnt signalling might be activated in *Usp9x* ^−/*Y*^ neocortices. However, the data indicate that any effect of Usp9x depletion on Wnt signalling is complex and cell context specific. While pβ-catenin33/37/41 levels were increased 6-fold in *Usp9x* ^−/*Y*^ neocortices, the transcriptionally active form Tyr654 pβ-catenin was only slightly elevated (Fig. 3F–H). Similarly, expression of Wnt target gene *Ccdn1* was clearly increased (by 60%) *in*-*vivo* but expression of *Axin2* was only marginally elevated (3%) (Fig. 5A,B). A similar, if not somewhat enhanced, dynamic range in *CCND1* (600%) and *AXIN2* (25%) expression was detected in the ReNcell VM human NP cell line (Fig. 5E,F). Induction of *CCND1* expression following USP9X depletion in ReNcell VM NPs was of a similar level as exposure to exogenous Wnt3a (Fig. 5H). Use of the TOPFlash reporter system demonstrated that depletion of USP9X is capable of directly activating Wnt transcription, to the same level as exogenous Wnt3a, in HEK293 cells (Fig. 5C). Further, USP9X acts cell autonomously as the external Wnt inhibitor DKK1 does not affect it.

However, while ingenuity pathway analysis identified the Wnt pathway as significantly affected, the detected transcripts were components of Wnt pathway rather than targets. In addition, *Ccnd1* is not an exclusive Wnt target gene but is regulated by numerous signalling pathways, including Notch^60, 61^.

In contrast to increased β-catenin levels, Numb and Itch protein levels decreased in *Usp9x* ^−/*Y*^ brains. The depletion of the E3 ubiquitin ligase Itch was not unexpected as Usp9x recuses it from auto-ubiquitylation and proteasomal degradation in multiple cell types^15^. However, the observation that Usp9x co-immunoprecipitates and co-localizes (Figs 7F–G, S9) with Numb in NPs, and Numb protein levels are clearly reduced in *Usp9x* ^−/*Y*^ neocortices (Fig. 7C–E) is novel. To our knowledge Usp9x is the first DUB shown to interact with, and maintain the levels of Numb, which is polyubiqutylated by the E3 ligases MDM2^51^ and LNX^52^. Numb recruits Itch to membrane-tethered Notch receptors^49^. Usp9x immunoprecipitates within this complex, suggesting Usp9x may influence its stability and/or function. As a consequence of Usp9x deletion, NICD accumulated in *Usp9x* ^−/*Y*^ cortices, and increased expression of Notch target genes. The slight but significantly increased *Hes5* expression paralleled that of the Wnt target *Axin2*, suggesting a partial activation of Notch signalling. However, an increase in the BLBP expression suggested even this partial activation was sufficient to propagate a physiological response in *Usp9x* ^−/*Y*^ NPs.

Here we present data that Usp9x interacts with three independent mechanisms regulating NP function, namely, polarised cell adhesion, Wnt and Notch signalling. To some extent Usp9x appears capable of directly regulating components of all three pathways. Deletion of Usp9x transiently disrupted AJs and polarity in NPs resulting in the premature appearance and ectopic localisation of Tbr2^+^ intermediate progenitors. The most dramatic effect of Usp9x loss was the increase in β-catenin protein levels, especially pβ-catenin33/37/41. This was possibly due to altered stoichiometry of the β-catenin destruction complex in the absence of Usp9x. Loss of Usp9x also activated the Wnt signalling pathway and, at least in cultured cells, this can occur directly. However, in NPs *in*-*vivo* the effects are more modest. This may be due to the context specific role of both Usp9x^5, 62^, and β-catenin transcriptional activation^63^ which are influenced by other signalling pathways. Indeed both Wnt and Notch activation increase *Ccdn1* expression^61, 64^ and this may explain the more robust activation of this gene. We also show for the first time that Usp9x interacts with the Notch inhibitor Numb in NPs and loss of Usp9x significantly decreased Numb and Itch protein levels.

The effect of Usp9x deletion on NPs on brain development was not as severe as inhibition of each pathway alone possibly reflecting the counteracting roles of Notch and Wnt in progenitor cells^40^ Nonetheless, missense mutations in USP9X and loss of heterozygosity give rise to multiple neurodevelopmental disorders in humans^65, 66^ and both USP9X gain of function and loss of function^67^ are tumorigenic in numerous cell types. The results presented here provide insight into some molecular mechanisms regulated by USP9X, which might be relevant to these pathologies. However, further experiments are warranted to elucidate how Usp9x integrates extrinsic pathways with cell adhesion and polarity.

## Material and Methods

### Animals

All animals were used and maintained in accordance with guidelines approved by the Griffith University Animal Ethics Committee under the animal ethics application BPS 02/11 and ESK06/14. The study followed the Australian Code of Practice for the Care and Use of Animals for Scientific Purposes. Usp9x conditionally deleted mice (*Usp9x* ^−/*Y*^) were generated by mating Usp9x^*loxP*^/^*loxP*^ female mice with Nestin-*Cre* male mice and the genotyping was conducted as described previously^6^.

### Cell culture

HEK293 cells were maintained in DMEM supplemented with 10% fetal calf serum at 37 °C in an incubator with 5% CO_2_. ReNcell VM was obtained from Millipore (Darmstadt, Germany) and maintained according to the manufactures instructions. Doxycycline-inducible Usp9x knock-down ReNcell VMs were generated as described previously^29, 68^.

### Immunohistochemistry

Time-mated mice were sacrificed by cervical dislocation; embryos were removed and dissected in ice-cold HBSS. Extracted embryonic heads were dropped fixed in 4% paraformaldehyde in PBS. The heads were then cryoprotected by immersion in 15% sucrose and 30% sucrose gradients and finally embedded in Tissue-Tek Optimal Cutting Temperature compound (OTC) (Sakura). 10 μm coronal sections were made using Leica cryostat and mounted on Super Frost Plus microscope slides (Thermo Fisher Scientific). The sections were permeabilized using 1% SDS for 4 minutes then blocked with 2% BSA for 30 minutes at room temperature. Primary and secondary antibodies diluted in 2% BSA were incubated overnight at 4 °C and 1h at RT respectively. The used antibodies and their dilutions were listed in the Table S4.

### Quantification

The cortex was defined as the area from the cortical hem to the pallial-subpallial boundary in each confocal image. Average NP and neuronal populations were measured as described^69^. Briefly, the VZ thickness was measured in a confocal image stained with SOX2 by first drawing a line at right angles to the ventricular surface, from the apical surface to the pial surface. The length of this line was measured by ImageJ 1.60s software (National Institute of Health) and considered total cortical thickness. A line drawn from the apical to basal side of the SOX2-positive band was considered as VZ thickness. The average NP population was calculated as a ratio between the VZ and cortex thicknesses at five different points along the rostral-caudal axis in each image and the mean value of this ratio was used for all statistical analysis. The same method was used for the DCX positive cortical plate to measure the average neuronal population.

### Immunoblot

Forebrains were dissected from extracted embryos, flash frozen with liquid Nitrogen and stored in -80 until use. Cortical tissues were lysed using lysis buffer (025 M Tris, 0.15 M NaCl, 0.001 M EDTA, 1% NP-40, 5% glycerol; pH 7.4) with 100 μl/mL protease and phosphatase inhibitor mix (Cell Signalling). Protein lysate was processed for immunoblotting and visualised with ECL substrate as described^6^. Antibodies used are listed in Table S5. Images of the full length immunoblots are presented in Supplementary data.

### Co-immunoprecipitation

Proteins were extracted from embryonic mouse cortical tissues by sonication in the presence of IP lysis buffer - 20 mM HEPES pH 7.8, 400 mM KCl, 5 mM EDTA, 0.4% NP40, 10% glycerol, 1 mM DTT, protease and phosphatase inhibitor cocktails (Cell Signalling). Immunoprecipitation was conducted using Pierce co-immunoprecipitation kit (Thermo Scientific) according to manufacturer’s instructions before being subjected to immunoblot analysis.

### Quantitative RT-PCR and Microarray

RNA was isolated from cells or embryonic tissue using Trizol reagent (Invitrogen). The cDNA was generated using 2 μg of isolated RNA using the High capacity Reverse transcription kit (Applied Biosystem) according to the manufacturer’s instructions. Quantitative real reactions were conducted on the Rotor gene 6000 (Corbett Life Science) using the SensiFAST HRM kit (Bioline). All samples were run in quadruples and normalised to GAPDH expression. Primer sequences listed in Table S6. For each genotype four embryos were used for microarray analyses. 500 ng of total RNA was processed using Ambion Illumina TotalPrep RNA amplification kit and cRNAs were hybridized to Illumina MouseRef-8 v2.0 Expression BeadChips (Illumina) and scanned on an Illumina Beadstation. Data was process and quantile normalised using R/BioConductor lumi package as described previously^70^. Differentially expressed transcripts were identified using the limma package - (-linear models for microarray^71^, at p = 0.05. Functional over-representation analysis was determined using right-tailed Fisher’s exact test using Ingenuity Pathway Analysis 8.5 (Ingenuity Systems). Microarray data was deposited into ArrayExpress (Accession Number E-MTAB-4150). Differentially expressed genes were clustered using Euclidean distance^65^ in Cluster 3.0 and visualized in Java Freeview^66^.

### Luciferase assay

USP9X expression was depleted in HEK293 cells using USP9X siRNA obtained from Millenium Science (SMARTpool ON-TARGETplus USP9x siRNA Cat# L-006099-00-0005) was used to knockdown USP9X. Scrambled siRNA (ON-TARGETplus Non-targeting Pool Cat# D-001810-10-05) was used as control. USP9X-depleted HEK293 cells were then transfected with plasmids pGL3-TOP and pGL3-FOP containing TCF luciferase-reporter construct^72^ using Lipofectamine 2000 reagent (Invitrogen). Wnt3A or DKK1 added to the cells 24 hours after the transfection. 1 hour after the Wnt3a (100 ng/µl) (R&D system Cat# 5036-WN) and DKK1 (100 ng/µl) (Sigma Cat# SRO3258) treatment, cells were lysed using luciferase lysis buffer (Promega). To measure the luciferase activity, 10 μl of cell lysate was transferred to a black 96-well Optiplate (Perkin Elmer). Luciferase activities were performed in triplicate.

### Statistical analysis

Statistical analysis was performed using Prism 5 (GraphPad Prism). For all analysis at least three biological repeats were assessed. Statistical analysis was perform using one-way ANOVA and Tukey’s post-hoc tests, for experiments containing more than two samples, or with a Student T-test for data sets with only two samples. The error bars on all the graphs represent SEM. All the values were presented as mean ± SEM.

## Electronic supplementary material

Supplementary Figures

Supplementary Tables

## References

[1] Taverna E, Gotz M, Huttner WB (2014). The cell biology of neurogenesis: toward an understanding of the development and evolution of the neocortex. Annual review of cell and developmental biology.

[2] Tuoc TC, Stoykova A (2010). Roles of the ubiquitin-proteosome system in neurogenesis. Cell cycle.

[3] Jolly LA, Taylor V, Wood SA (2009). USP9X enhances the polarity and self-renewal of embryonic stem cell-derived neural progenitors. Molecular biology of the cell.

[4] Friocourt G (2005). Doublecortin interacts with the ubiquitin protease DFFRX, which associates with microtubules in neuronal processes. Molecular and cellular neurosciences.

[5] Murtaza M, Jolly LA, Gecz J, Wood SA (2015). La FAM fatale: USP9X in development and disease. Cellular and molecular life sciences: CMLS.

[6] Stegeman S (2013). Loss of Usp9x disrupts cortical architecture, hippocampal development and TGFbeta-mediated axonogenesis. PloS one.

[7] Taya S (1998). The Ras target AF-6 is a substrate of the fam deubiquitinating enzyme. J Cell Biol.

[8] Theard D (2010). USP9x-mediated deubiquitination of EFA6 regulates de novo tight junction assembly. The EMBO journal.

[9] Murray RZ, Jolly LA, Wood SA (2004). The FAM deubiquitylating enzyme localizes to multiple points of protein trafficking in epithelia, where it associates with E-cadherin and beta-catenin. Molecular biology of the cell.

[10] Al-Hakim AK (2008). Control of AMPK-related kinases by USP9X and atypical Lys(29)/Lys(33)-linked polyubiquitin chains. The Biochemical journal.

[11] Al-Hakim AK (2005). 14-3-3 cooperates with LKB1 to regulate the activity and localization of QSK and SIK. Journal of cell science.

[12] Stegmuller J, Bonni A (2010). Destroy to create: E3 ubiquitin ligases in neurogenesis. F1000 Biol Rep.

[13] Choe EA (2007). Neuronal morphogenesis is regulated by the interplay between cyclin-dependent kinase 5 and the ubiquitin ligase mind bomb 1. The Journal of neuroscience: the official journal of the Society for Neuroscience.

[14] Fischer-Vize JA, Rubin GM, Lehmann R (1992). The fat facets gene is required for Drosophila eye and embryo development. Development.

[15] Mouchantaf R (2006). The ubiquitin ligase itch is auto-ubiquitylated *in vivo* and *in vitro* but is protected from degradation by interacting with the deubiquitylating enzyme FAM/USP9X. The Journal of biological chemistry.

[16] Taya S, Yamamoto T, Kanai-Azuma M, Wood SA, Kaibuchi K (1999). The deubiquitinating enzyme Fam interacts with and stabilizes beta-catenin. Genes to cells: devoted to molecular & cellular mechanisms.

[17] Li (2012). Wnt signaling through inhibition of beta-catenin degradation in an intact Axin1 complex. Cell.

[18] Major MB (2007). Wilms tumor suppressor WTX negatively regulates WNT/beta-catenin signaling. Science.

[19] Pantaleon M (2001). FAM deubiquitylating enzyme is essential for preimplantation mouse embryo development. Mechanisms of development.

[20] Katayama K (2011). Loss of RhoA in neural progenitor cells causes the disruption of adherens junctions and hyperproliferation. Proceedings of the National Academy of Sciences of the United States of America.

[21] Gotz M, Huttner WB (2005). The cell biology of neurogenesis. Nature reviews. Molecular cell biology.

[22] Cappello S (2006). The Rho-GTPase cdc42 regulates neural progenitor fate at the apical surface. Nature neuroscience.

[23] Leone DP, Srinivasan K, Brakebusch C, McConnell SK (2010). The rho GTPase Rac1 is required for proliferation and survival of progenitors in the developing forebrain. Developmental neurobiology.

[24] Valenta T, Hausmann G, Basler K (2012). The many faces and functions of beta-catenin. The EMBO journal.

[25] Lyashenko N (2011). Differential requirement for the dual functions of beta-catenin in embryonic stem cell self-renewal and germ layer formation. Nature cell biology.

[26] Roura S, Miravet S, Piedra J, Garcia de Herreros A, Dunach M (1999). Regulation of E-cadherin/Catenin association by tyrosine phosphorylation. The Journal of biological chemistry.

[27] Ouyang W (2016). beta-catenin is regulated by USP9x and mediates resistance to TRAIL-induced apoptosis in breast cancer. Oncology reports.

[28] Gerlach JP, Emmink BL, Nojima H, Kranenburg O, Maurice MM (2014). Wnt signalling induces accumulation of phosphorylated beta-catenin in two distinct cytosolic complexes. Open biology.

[29] Bridges CR (2017). USP9X deubiquitylating enzyme maintains RAPTOR protein levels, mTORC1 signalling and proliferation in neural progenitors. Scientific reports.

[30] Liu H (2015). WP1130 increases doxorubicin sensitivity in hepatocellular carcinoma cells through usp9x-dependent p53 degradation. Cancer letters.

[31] Kushwaha D (2015). USP9X inhibition promotes radiation-induced apoptosis in non-small cell lung cancer cells expressing mid-to-high MCL1. Cancer biology & therapy.

[32] Kapuria V (2010). Deubiquitinase inhibition by small-molecule WP1130 triggers aggresome formation and tumor cell apoptosis. Cancer Res.

[33] Ma P (2014). The ubiquitin ligase RNF220 enhances canonical Wnt signaling through USP7-mediated deubiquitination of beta-catenin. Molecular and cellular biology.

[34] Yun SI (2015). Ubiquitin specific protease 4 positively regulates the WNT/beta-catenin signaling in colorectal cancer. Molecular oncology.

[35] Shi J (2015). Deubiquitinase USP47/UBP64E Regulates beta-Catenin Ubiquitination and Degradation and Plays a Positive Role in Wnt Signaling. Molecular and cellular biology.

[36] Lui TT (2011). The ubiquitin-specific protease USP34 regulates axin stability and Wnt/beta-catenin signaling. Molecular and cellular biology.

[37] Machon O (2007). A dynamic gradient of Wnt signaling controls initiation of neurogenesis in the mammalian cortex and cellular specification in the hippocampus. Developmental biology.

[38] Chenn A, Walsh CA (2002). Regulation of cerebral cortical size by control of cell cycle exit in neural precursors. Science.

[39] Woodhead GJ, Mutch CA, Olson EC, Chenn A (2006). Cell-autonomous beta-catenin signaling regulates cortical precursor proliferation. The Journal of neuroscience: the official journal of the Society for Neuroscience.

[40] Mutch CA, Funatsu N, Monuki ES, Chenn A (2009). Beta-catenin signaling levels in progenitors influence the laminar cell fates of projection neurons. The Journal of neuroscience: the official journal of the Society for Neuroscience.

[41] Kwon C (2011). Notch post-translationally regulates beta-catenin protein in stem and progenitor cells. Nature cell biology.

[42] Mossinger J (2012). Phosphatidylinositol 4-kinase IIalpha function at endosomes is regulated by the ubiquitin ligase Itch. EMBO reports.

[43] Wei W, Li M, Wang J, Nie F, Li L (2012). The E3 ubiquitin ligase ITCH negatively regulates canonical Wnt signaling by targeting dishevelled protein. Molecular and cellular biology.

[44] Zhang J, Liu M, Su Y, Du J, Zhu AJ (2012). A targeted *in vivo* RNAi screen reveals deubiquitinases as new regulators of Notch signaling. G3.

[45] Basak O, Taylor V (2007). Identification of self-replicating multipotent progenitors in the embryonic nervous system by high Notch activity and Hes5 expression. The European journal of neuroscience.

[46] Hitoshi S (2002). Notch pathway molecules are essential for the maintenance, but not the generation, of mammalian neural stem cells. Genes & development.

[47] Anthony TE, Mason HA, Gridley T, Fishell G, Heintz N (2005). Brain lipid-binding protein is a direct target of Notch signaling in radial glial cells. Genes & development.

[48] Mertz J (2015). Sequential Elution Interactome Analysis of the Mind Bomb 1 Ubiquitin Ligase Reveals a Novel Role in Dendritic Spine Outgrowth. Molecular & cellular proteomics: MCP.

[49] McGill MA, McGlade CJ (2003). Mammalian numb proteins promote Notch1 receptor ubiquitination and degradation of the Notch1 intracellular domain. The Journal of biological chemistry.

[50] Rasin MR (2007). Numb and Numbl are required for maintenance of cadherin-based adhesion and polarity of neural progenitors. Nature neuroscience.

[51] Sczaniecka M (2012). MDM2 protein-mediated ubiquitination of numb protein: identification of a second physiological substrate of MDM2 that employs a dual-site docking mechanism. The Journal of biological chemistry.

[52] Nie J (2002). LNX functions as a RING type E3 ubiquitin ligase that targets the cell fate determinant Numb for ubiquitin-dependent degradation. The EMBO journal.

[53] Lin D (2000). A mammalian PAR-3-PAR-6 complex implicated in Cdc42/Rac1 and aPKC signalling and cell polarity. Nature cell biology.

[54] Imai F (2006). Inactivation of aPKClambda results in the loss of adherens junctions in neuroepithelial cells without affecting neurogenesis in mouse neocortex. Development.

[55] Srinivasan K (2008). MALS-3 regulates polarity and early neurogenesis in the developing cerebral cortex. Development.

[56] Chalasani K, Brewster RM (2011). N-cadherin-mediated cell adhesion restricts cell proliferation in the dorsal neural tube. Molecular biology of the cell.

[57] Mutch CA, Schulte JD, Olson E, Chenn A (2010). Beta-catenin signaling negatively regulates intermediate progenitor population numbers in the developing cortex. PloS one.

[58] Gao C (2014). Induction of Gsk3beta-beta-TrCP interaction is required for late phase stabilization of beta-catenin in canonical Wnt signaling. The Journal of biological chemistry.

[59] Yang J (2006). Adenomatous polyposis coli (APC) differentially regulates beta-catenin phosphorylation and ubiquitination in colon cancer cells. The Journal of biological chemistry.

[60] Cohen B (2010). Cyclin D1 is a direct target of JAG1-mediated Notch signaling in breast cancer. Breast cancer research and treatment.

[61] Ling H, Jolicoeur P (2013). Notch-1 signaling promotes the cyclinD1-dependent generation of mammary tumor-initiating cells that can revert to bi-potential progenitors from which they arise. Oncogene.

[62] Nanayakkara DM, Nguyen MN, Wood SA (2016). Deubiquitylating enzyme, USP9X, regulates proliferation of cells of head and neck cancer lines. Cell proliferation.

[63] Nakamura Y, de P Alves E, Veenstra GJ, Hoppler S (2016). Tissue- and stage-specific Wnt target gene expression is controlled subsequent to beta-catenin recruitment to cis-regulatory modules. Development.

[64] Das D (2010). Notch induces cyclin-D1-dependent proliferation during a specific temporal window of neural differentiation in ES cells. Developmental biology.

[65] de Hoon MJ, Imoto S, Nolan J, Miyano S (2004). Open source clustering software. Bioinformatics.

[66] Saldanha AJ (2004). Java Treeview–extensible visualization of microarray data. Bioinformatics.

[67] Perez-Mancera, P. A. *et al*. The deubiquitinase USP9X suppresses pancreatic ductal adenocarcinoma. *Nature* **486**, 266–270, doi:10.1038/nature11114 (2012). 10.1038/nature11114 PMC3376394 22699621

[68] Brown, C. Y. *et al*. Robust, Reversible Gene Knockdown Using a Single Lentiviral Short Hairpin RNA Vector. *Human Gene Therapy* **21**, 1005–1017 (2010). 10.1089/hum.2009.107 20615123

[69] Haubst, N. *et al*. Molecular dissection of Pax6 function: the specific roles of the paired domain and homeodomain in brain development. *Development* **131**, 6131–6140, doi:10.1242/dev.01524 (2004). 10.1242/dev.01524 15548580

[70] Cook, A. L. *et al.* NRF2 activation restores disease related metabolic deficiencies in olfactory neurosphere-derived cells from patients with sporadic Parkinson’s disease. *PloS one* **6**, e21907, doi:10.1371/journal.pone.0021907 (2011). 10.1371/journal.pone.0021907 PMC3128624 21747966

[71] Smyth, G. K. In Bioinformatics and Computational Biology Solutions Using R and Bioconductor Statistics for Biology and Health (eds Robert Gentleman *et al.*) Ch. **23**, 397–420 (Springer New York, 2005).

[72] Molenaar, M. *et al*. XTcf-3 transcription factor mediates beta-catenin-induced axis formation in Xenopus embryos. *Cell* **86**, 391–399 (1996). 10.1016/s0092-8674(00)80112-9 8756721

