# Supplementary material for: Loss of Usp9x disrupts cell adhesion, and components of the Wnt and Notch signaling pathways in neural progenitors
Source: Sci Rep. 2017 Aug 14;7:8109. doi: 10.1038/s41598-017-05451-5 (PMC5556043; doi:10.1038/s41598-017-05451-5)
Supplement: Supplementary file 1 — Supplementary Figures [file 41598_2017_5451_MOESM1_ESM.pdf]

Supplementary Information

Title: "Loss of Usp9x disrupts cell adhesion, and components of the Wnt and Notch signaling pathways in neural progenitors."

Authors: Susitha Premarathne, Mariyam Murtaza, Nicholas Matigian, Lachlan A Jolly and Stephen A Wood

Supplementary Figures

FigS1. Reduced N-cadherin and AF-6 immunoreactivity in E12.5 *Usp9x*$^{-/Y}$ neocortices. **(A)** Coronal sections of a further two independent E12.5 *Usp9x*$^{+/Y}$ and *Usp9x*$^{-/Y}$ neocortices stained for N-cadherin and AF-6 (in addition to Fig.1A). Compared to *Usp9x*$^{+/Y}$ (a, c, e) N-cadherins immunoreactivity was clearly reduced at the apical surface of *Usp9x*$^{-/Y}$ VZ (white arrows) (b, d, f). **(B)** Coronal sections of two independent E12.5 *Usp9x*$^{+/Y}$ and *Usp9x*$^{-/Y}$ neocortices stained for AF-6. Compared to *Usp9x*$^{+/Y}$ (a, c, e) AF-6 immunoreactivity was clearly reduced at the apical surface of *Usp9x*$^{-/Y}$ VZ (white arrows) (b, d, f). Scale bars = 80 μm. LV- lateral ventricle, VZ- ventricular zone.

FigS2. Heat map showing 1047 genes differently expressed between E12.5 *Usp9x*$^{+/Y}$ and *Usp9x*$^{-/Y}$ neocortices. Expression intensity of E12.5 Usp9x-/Y compared to littermate controls (n=4 for each genotype).

FigS3. Heat map showing 1397 genes differently expressed between E14.5 *Usp9x*$^{+/Y}$ and *Usp9x*$^{-/Y}$ neocortices. Expression intensity of E14.5 Usp9x-/Y compared to littermate controls (n=4 for each genotype).

FigS3. Neurogenesis is intact in *Usp9x*$^{-/Y}$ neocortices. **(A)** Tbr1^{+} neuronal expression in *Usp9x*$^{-/Y}$ neocortices. E12.5 (a, b), E14.5 (c, d) and E16.5 (e, f) neocortices stained with Tbr1 antibodies (representative of n=3 for each embryonic stage). **(B)** The graph represents the number of Tbr1^{+} cells expressed per unit area of neocortex. No difference was observed between *Usp9x*$^{+/Y}$ and *Usp9x*$^{-/Y}$ neocortices at any stage. **(C)** E12.5 (a, b), E14.5 (c, d), E16.5 (e, f) and E18.5 (g, h) neocortices stained with Dcx antibodies (n=3 for each embryonic stage). Dotted lines demark the borders of the ventricular zone and cortical plate. **(D)** The graph represents Dcx expression as the ratio of Dcx^{+} cortical plate thickness (orange double headed arrow) over total cortical thickness (red double headed arrow) (n=3). No difference was observed for Dcx^{+} neuroblasts expression between *Usp9x*$^{+/Y}$ and *Usp9x*$^{-/Y}$ neocortices in all the tested embryonic stages. Scale bars = 80 μm. LV- lateral ventricle, VZ- ventricular zone.

FigS4. Apoptosis in *Usp9x$^{-/Y}$* neocortices. (A) Cleaved Caspase 3 stained *Usp9x$^{-/Y}$* brains. E12.5 (a, b), E14.5 (c, d), E16.5 (e, f) and E18.5 (g, h) neocortices stained with cleaved caspase 3 antibodies (n=3 for each embryonic stage). Caspase staining failed to detect apoptotic cells in *Usp9x$^{+/Y}$* and *Usp9x$^{-/Y}$* brains at the tested embryonic stages. **(B)** TUNEL assay of *Usp9x$^{-/Y}$* brains. TUNEL assay on sections from E12.5 (a, b), E14.5 (c, d), E16.5 (f, g) and E18.5 (i, j) neocortices stained (n=3 for each embryonic stage). (e, h) Brain sections exposed to DNase1 were used as the positive controls. No TUNEL positive cells were observed at any tested embryonic stages. Scale bars = 80 µm. LV- lateral ventricle, VZ- ventricular zone.

FigS5. (A) Co-immunoprecipitation of N-cadherin from E12.5 *Usp9x$^{+/Y}$* and *Usp9x$^{-/Y}$* cortical lysates, with anti-β-catenin antibodies. Relative to inputs, similar amounts of N-cadherin were pulled down from *Usp9x$^{+/Y}$* and *Usp9x$^{-/Y}$* lysates suggesting the catenin-cadherin heterodimer is intact in E12.5 *Usp9x$^{-/Y}$* NPs. **(B-E)** Immunoblot analysis of total APC protein levels in *Usp9x$^{+/Y}$* and *Usp9x$^{-/Y}$* cortices at E12.5 and E14.5 (n=3). (C, E) No difference was observed for total APC protein levels between *Usp9x$^{+/Y}$* and *Usp9x$^{-/Y}$* brains at E12.5 and E14.5. **(F-I)** Immunoblot analysis of GSK-3β protein levels in *Usp9x$^{+/Y}$* and *Usp9x$^{-/Y}$* cortices at E12.5 and E14.5 (n=3). (G, I) No difference was observed for total GSK-3β protein levels between *Usp9x$^{+/Y}$* and *Usp9x$^{-/Y}$* cortices at E12.5 and E14.5. **(J)** USP9X protein is depleted and Ser33/37/ Thr41 pβ-catenin protein is increased in HEK293 cells 72 hours after siRNA transfection. β-tubulin used as loading control.

FigS6. qRT-PCR analyses of the dysregulated Wnt pathway genes identified by Ingenuity pathway analysis. (a-f) qRT-PCR analyses results of dysregulated Wnt signaling genes in E12.5 *Usp9x$^{-/Y}$* cortices. Expression of all the tested genes was significantly altered in *Usp9x$^{-/Y}$* cortices. (g-n) qRT-PCR analyses results of some of the dysregulated Wnt signaling genes in E14.5 *Usp9x$^{-/Y}$* cortices. Only Sox4 and Sox1 expression are significantly different in *Usp9x$^{-/Y}$* cortices. *, $p < 0.05$, **,$p<0.01$, ***, $p<0.001$

FigS7. Astroglial differentiation is normal in the *Usp9x*$^{-/Y}$ neocortex. (A) Coronal sections E12.5 *Usp9x*$^{+/Y}$ and *Usp9x*$^{-/Y}$ neocortices co-stained with BLBP and the astrocytic marker GFAP. No difference was observed for the GFAP expression (a, b) between *Usp9x*$^{+/Y}$ and *Usp9x*$^{-/Y}$ brains, despite increased BLBP expression (c, d) in *Usp9x*$^{-/Y}$ brains. **(B)** Similar pattern of GFAP (g, h) and BLBP (i, j) staining in E18.5 *Usp9x*$^{+/Y}$ and *Usp9x*$^{-/Y}$ brains. **(C, D)** *HES5* and *HES1* gene expression were significantly increased in USP9X-depeleted ReNcell VM NPs. Scale bars = 80 µm. LV- lateral ventricle, VZ- ventricular zone. *, $p < 0.05$.

FigS8. Mindbomb1 expression in *Usp9x*$^{-/Y}$ neocortices. (A, C) Immunoblot analysis revealed Mindbomb1 protein levels are unaltered in E12.5 and E14.5 *Usp9x*$^{-/Y}$ neocortices (n=3). **(B, D)** Quantification of Mindbomb1 protein levels relative to β-tubulin.

FigS9. **Usp9x co-localises with Numb in embryonic mouse brains.** Coronal sections of E14.5 neocortices immunostained with Usp9x (red arrows) and Numb (green arrows) antibodies. Yellow arrows show the extensive co-localization of Usp9x and Numb proteins most notably at the VZ.

Table S1. Microarray was conducted using cortical tissues of E12.5 *Usp9x*$^{+/Y}$ and *Usp9x*$^{-/Y}$ neocortices (n=4). Differentially expressed genes were annotated using the functional annotation tool of Ingenuity pathway analysis software.

Table S2. Microarray was conducted using cortical tissues of E14.5 *Usp9x*$^{+/Y}$ and *Usp9x*$^{-/Y}$ neocortices (n=4). Differentially expressed genes were annotated using the functional annotation tool of Ingenuity pathway analysis software.

Table S3. 147 genes commonly expressed between E14.5 and E12.5 U*sp9x*$^{-/Y}$ brains.

Table S4. Ingenuity pathway analysis identified Wnt signalling as one of the most significantly affected pathway in *Usp9x*$^{-/Y}$ neocortices (n=4).

Table S5. Details of the antibodies and antibody dilutions used for IHC analysis.

Table S6. Details of the antibodies and antibody dilutions used for IB analysis.

Table S7. Primer sequences.

A

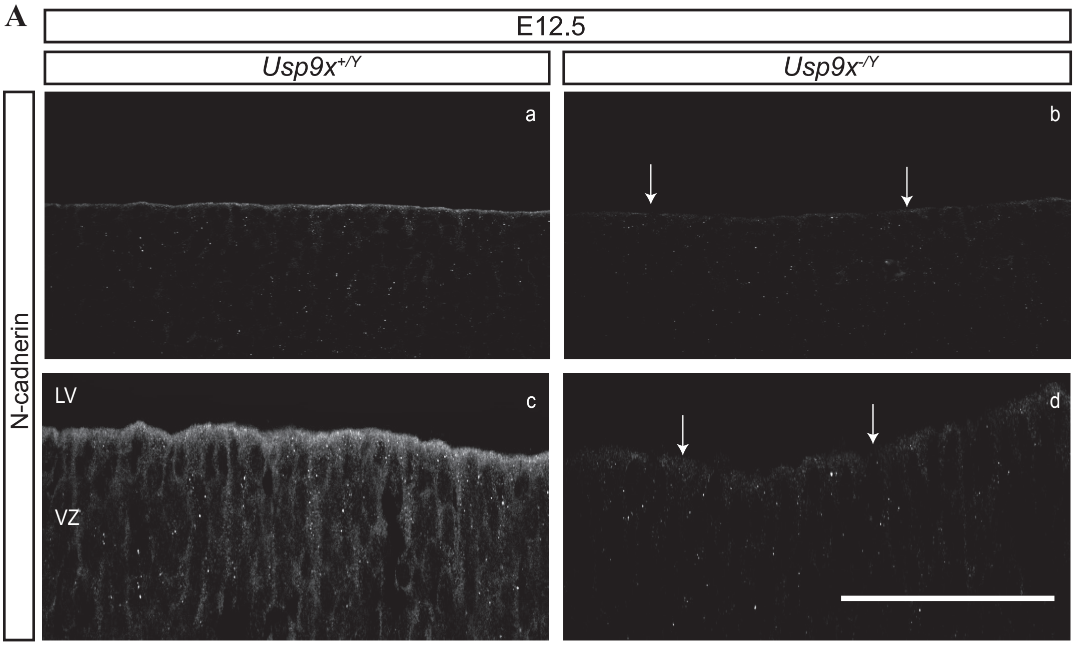
E12.5
Usp9x+/Y
Usp9x-/Y
N-cadherin
a
b
LV
c
VZ
d

B

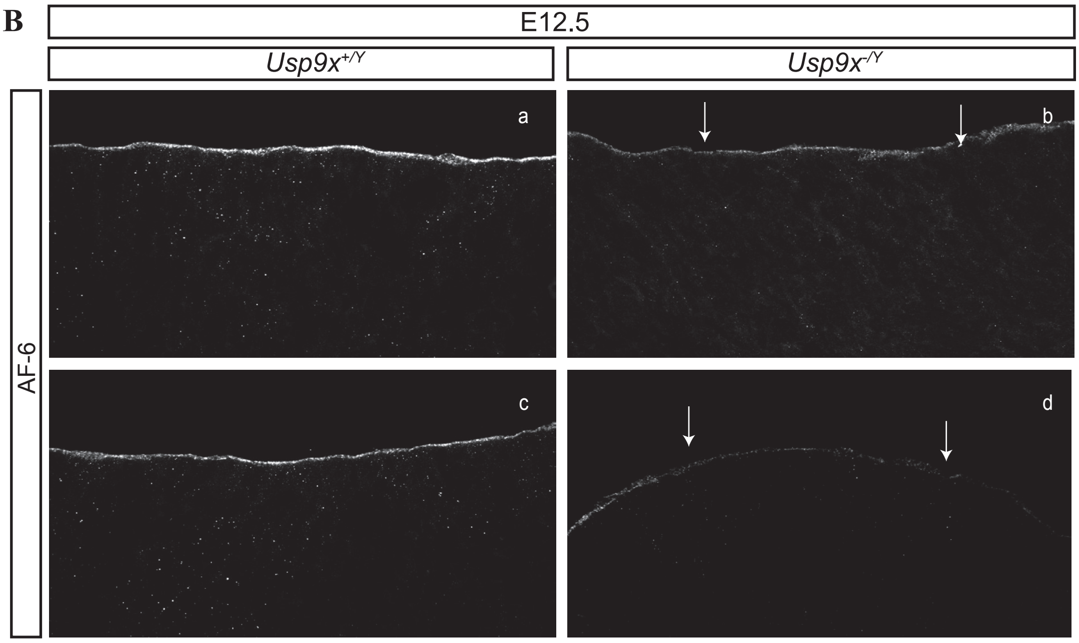
E12.5
Usp9x+/Y
Usp9x-/Y
AF-6
a
b
c
d

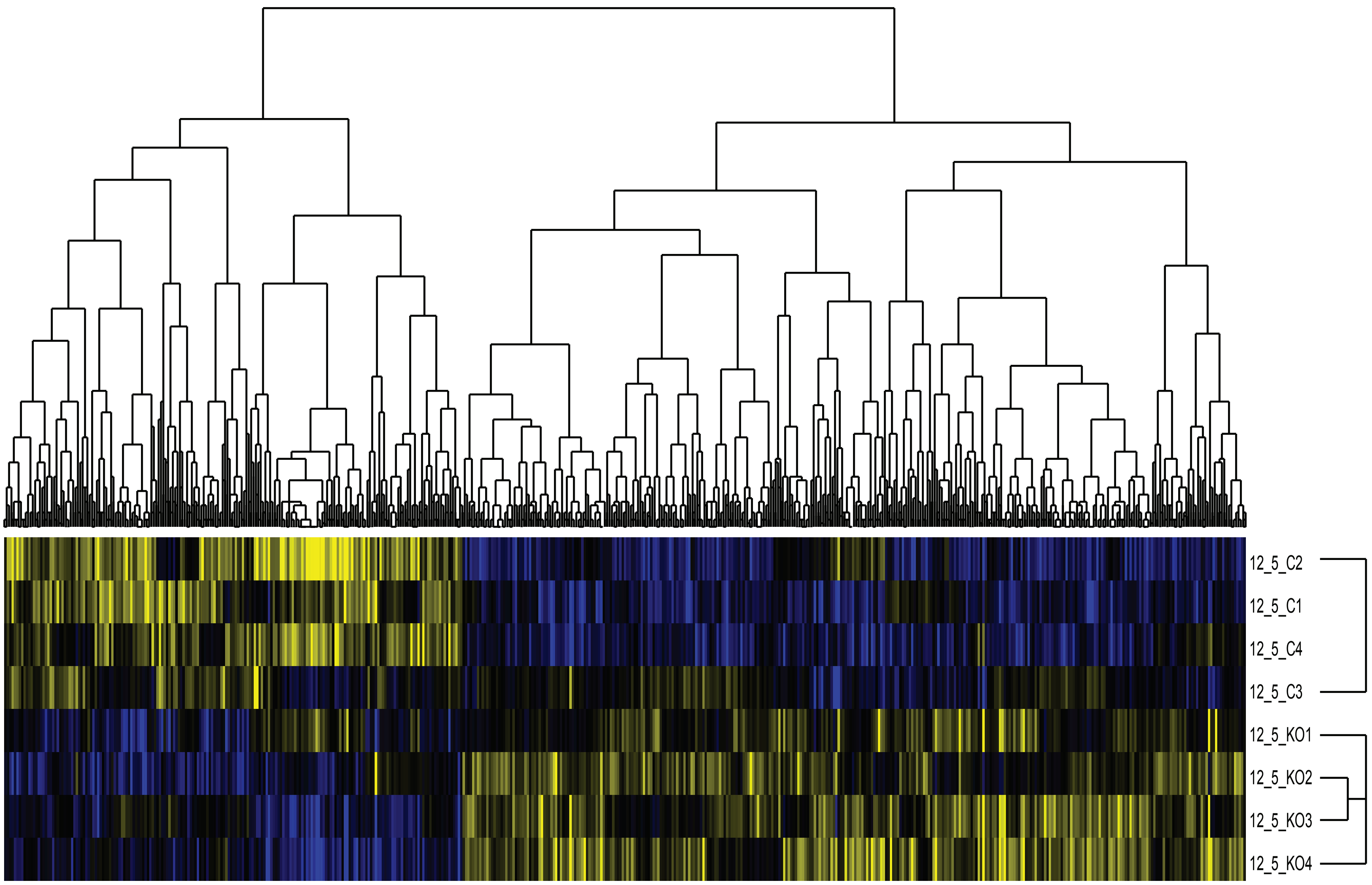
12_5_C2
12_5_C1
12_5_C4
12_5_C3
12_5_KO1
12_5_KO2
12_5_KO3
12_5_KO4

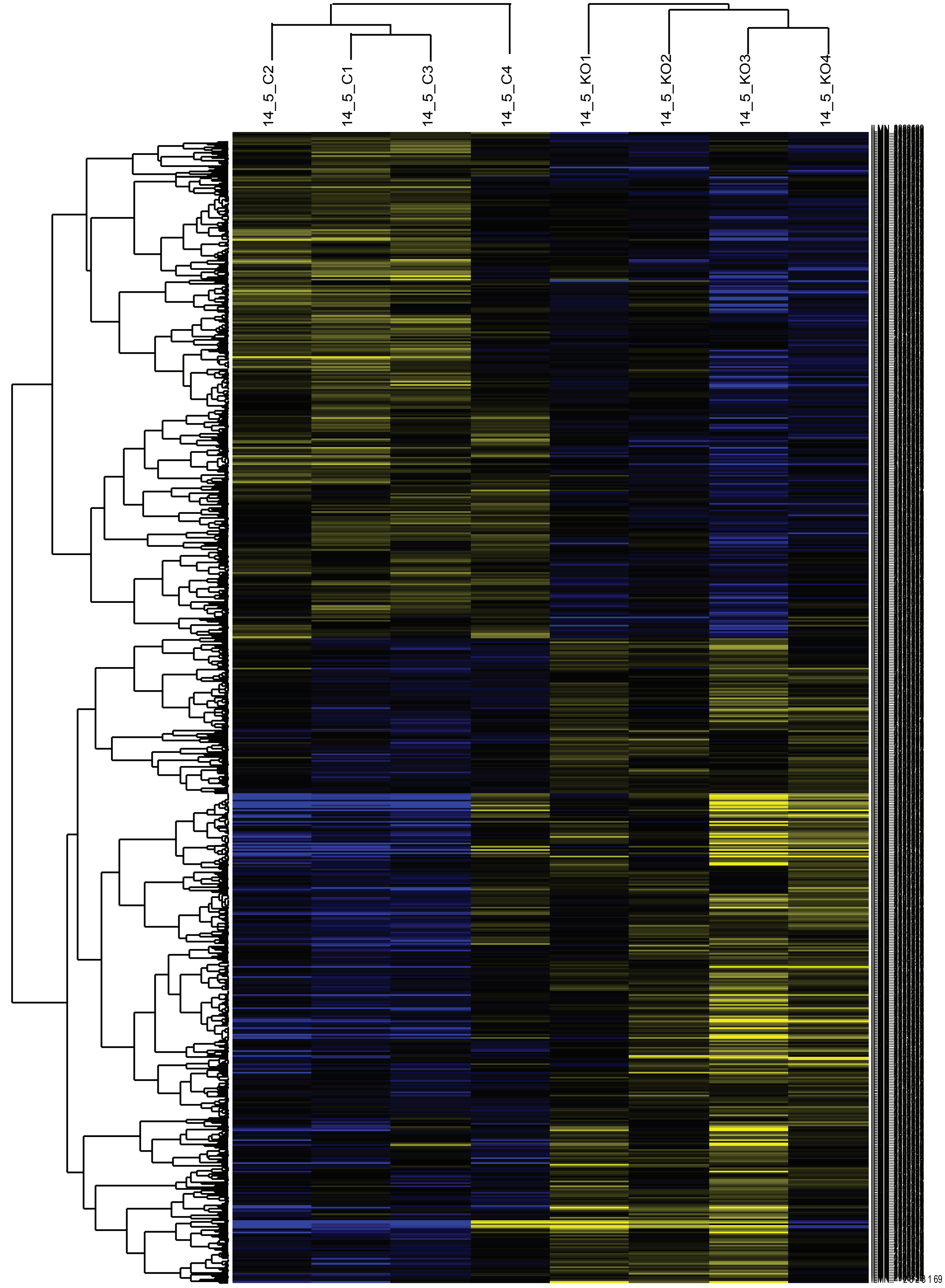

14_5_C2
14_5_C1
14_5_C3
14_5_C4
14_5_KO1
14_5_KO2
14_5_KO3
14_5_KO4

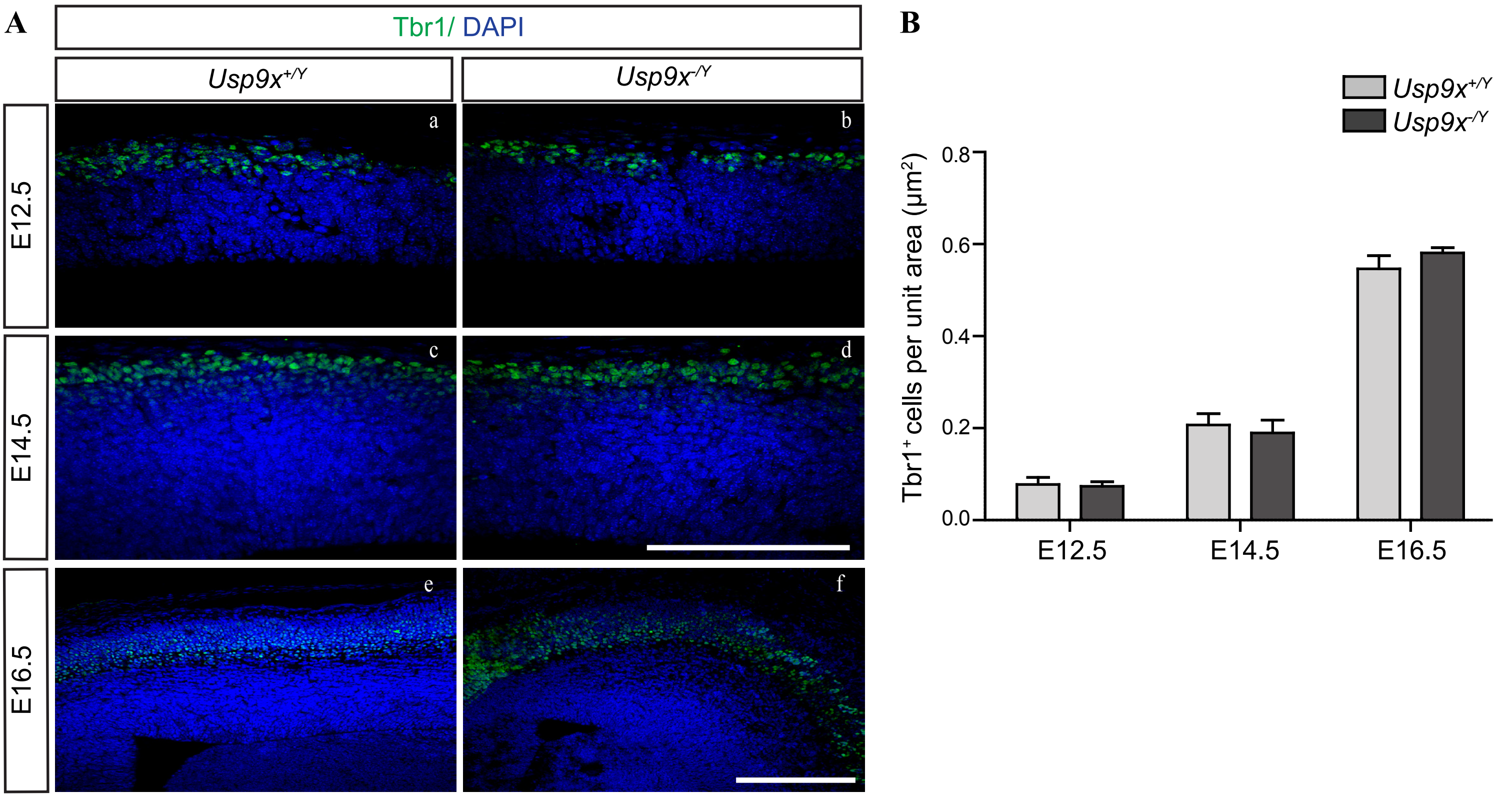
A
Tbr1/ DAPI
Usp9x+/Y
Usp9x-/Y
E12.5
E14.5
E16.5
a
b
c
d
e
f
B
Usp9x+/Y
Usp9x-/Y
Tbr1+ cells per unit area (μm2)
0.8
0.6
0.4
0.2
0.0
E12.5
E14.5
E16.5

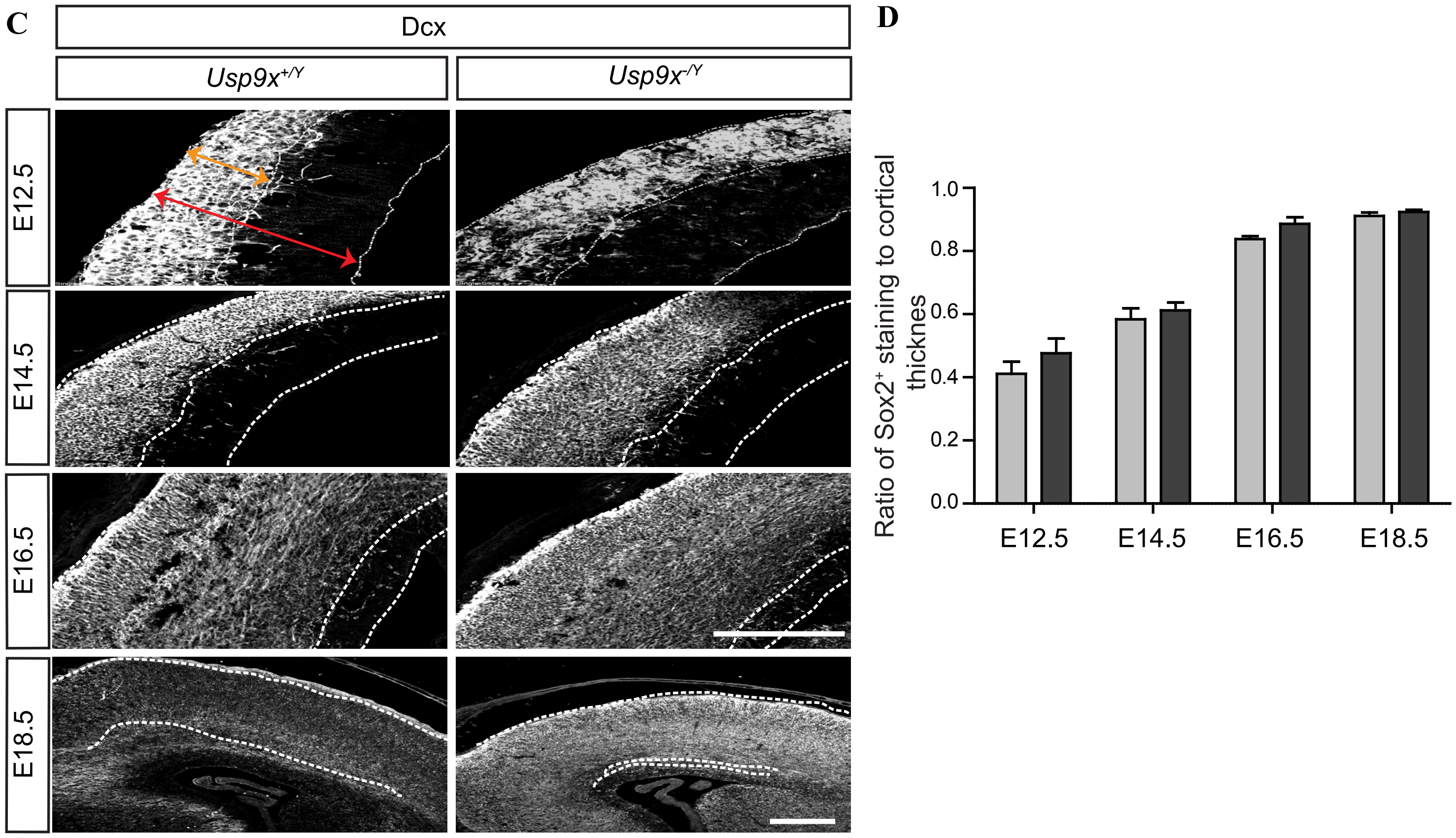
C
Dcx
Usp9x+/Y
Usp9x-/Y
E12.5
E14.5
E16.5
E18.5
D
Ratio of Sox2+ staining to cortical thicknes
1.0
0.8
0.6
0.4
0.2
0.0
E12.5
E14.5
E16.5
E18.5

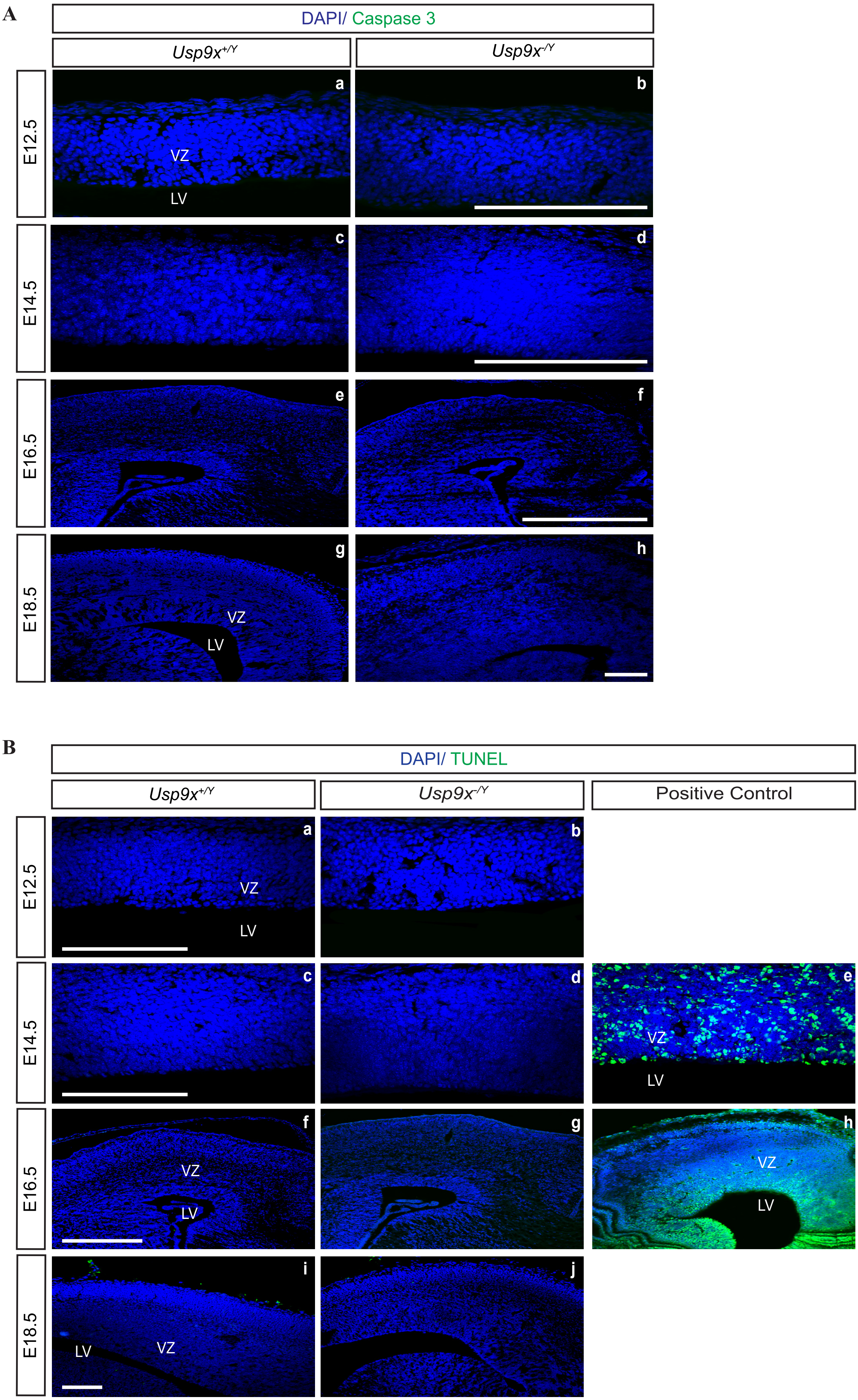
A
DAPI/ Caspase 3
Usp9x+/Y
Usp9x-/Y
E12.5
E14.5
E16.5
E18.5
a
b
c
d
e
f
g
h
VZ
LV
B
DAPI/ TUNEL
Usp9x+/Y
Usp9x-/Y
Positive Control
i
j

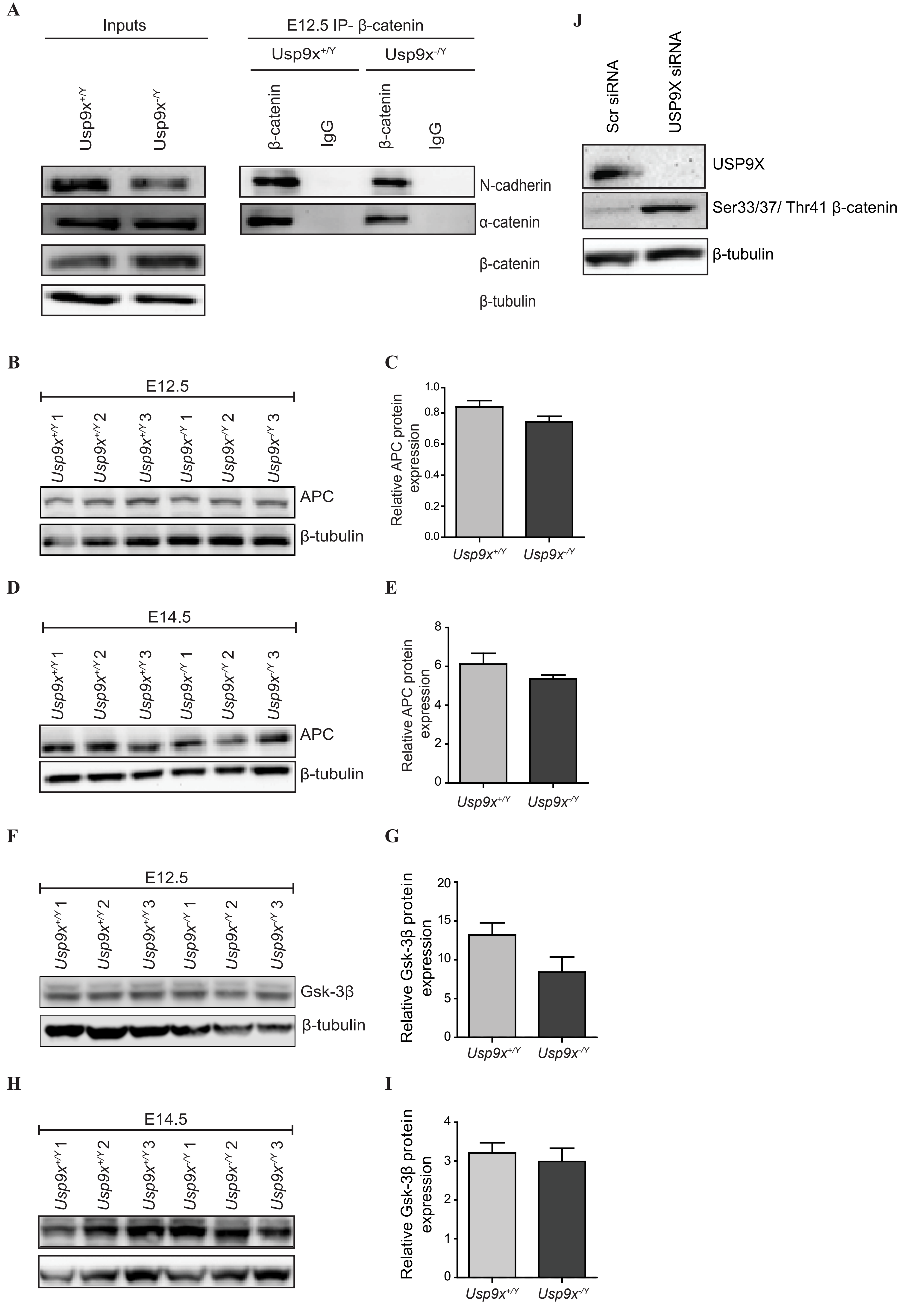
A
Inputs
E12.5 IP- β-catenin
Usp9x+/Y
Usp9x-/Y
β-catenin
IgG
N-cadherin
α-catenin
β-catenin
β-tubulin
J
Scr siRNA
USP9X siRNA
USP9X
Ser33/37/ Thr41 β-catenin
β-tubulin
B
E12.5
Usp9x+/Y 1
Usp9x+/Y 2
Usp9x+/Y 3
Usp9x-/Y 1
Usp9x-/Y 2
Usp9x-/Y 3
APC
β-tubulin
C
Relative APC protein expression
0.0
0.2
0.4
0.6
0.8
1.0
D
E14.5
APC
β-tubulin
E
Relative APC protein expression
0
2
4
6
8
F
E12.5
Gsk-3β
β-tubulin
G
Relative Gsk-3β protein expression
0
5
10
15
20
H
E14.5
I
Relative Gsk-3β protein expression
0
1
2
3
4

E12.5

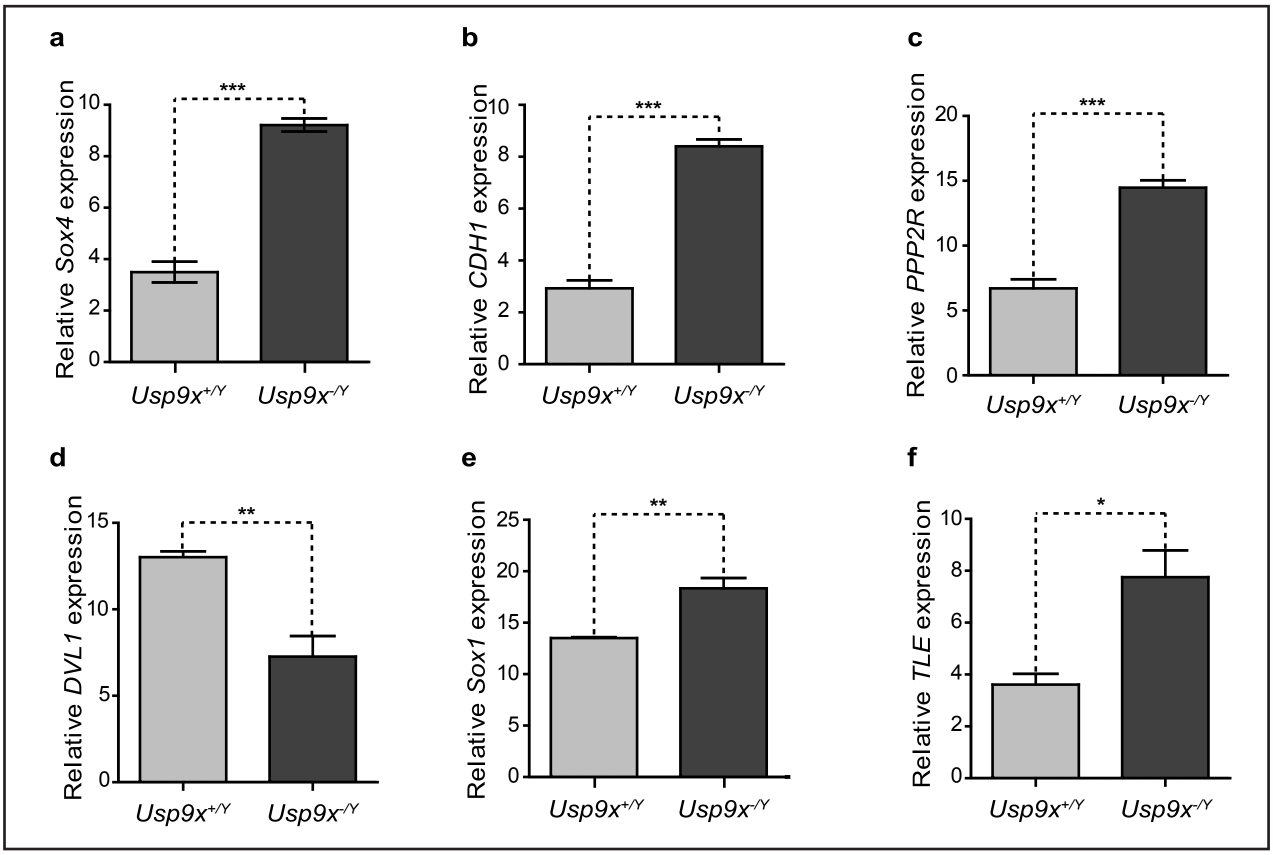
a
Relative Sox4 expression
***
b
Relative CDH1 expression
***
c
Relative PPP2R expression
***
d
Relative DVL1 expression
**
e
Relative Sox1 expression
**
f
Relative TLE expression
*
Usp9x+/Y
Usp9x-/Y

E14.5

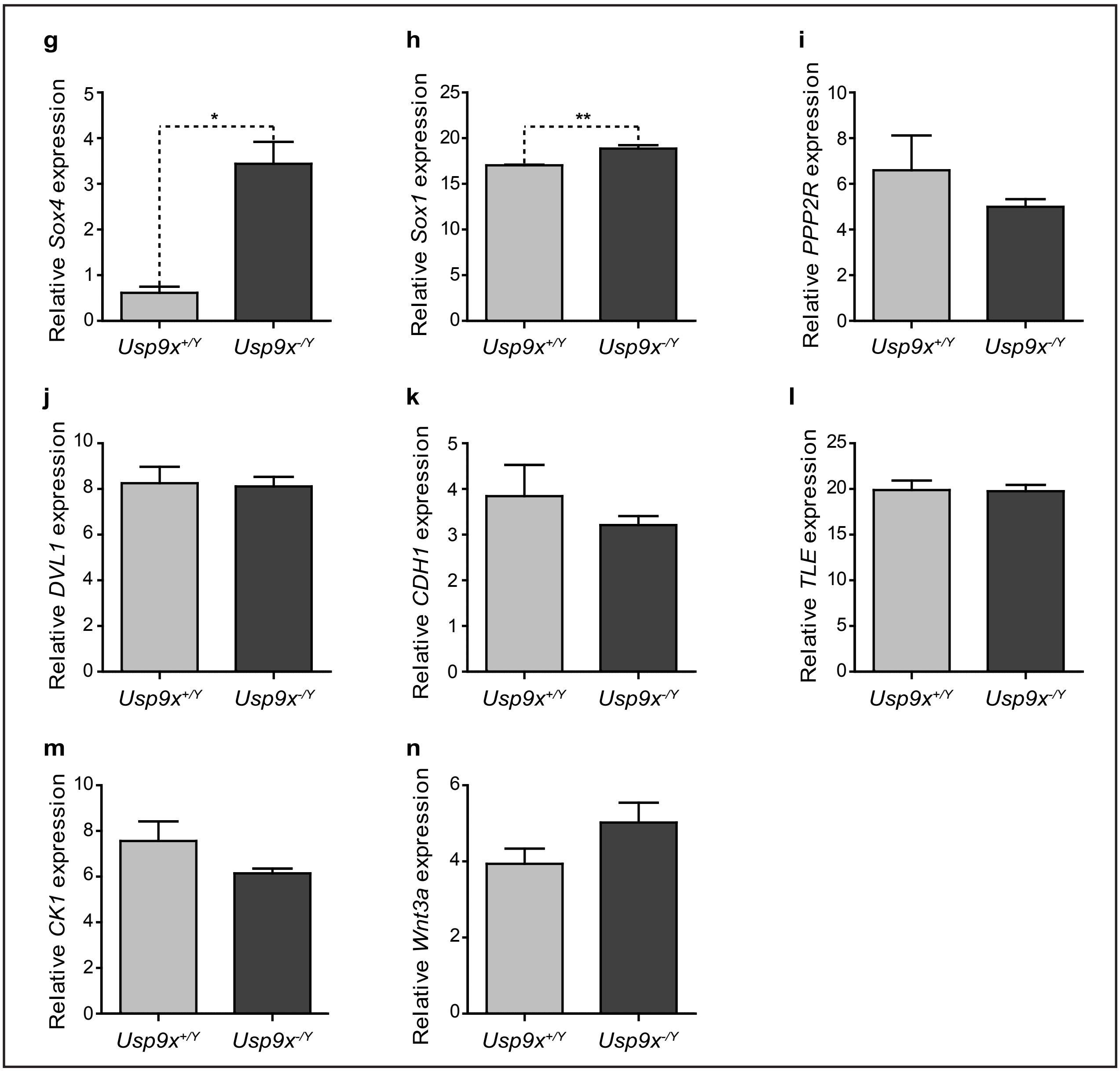
g
Relative Sox4 expression
*
h
Relative Sox1 expression
**
i
Relative PPP2R expression
j
Relative DVL1 expression
k
Relative CDH1 expression
l
Relative TLE expression
m
Relative CK1 expression
n
Relative Wnt3a expression
Usp9x+/Y
Usp9x-/Y

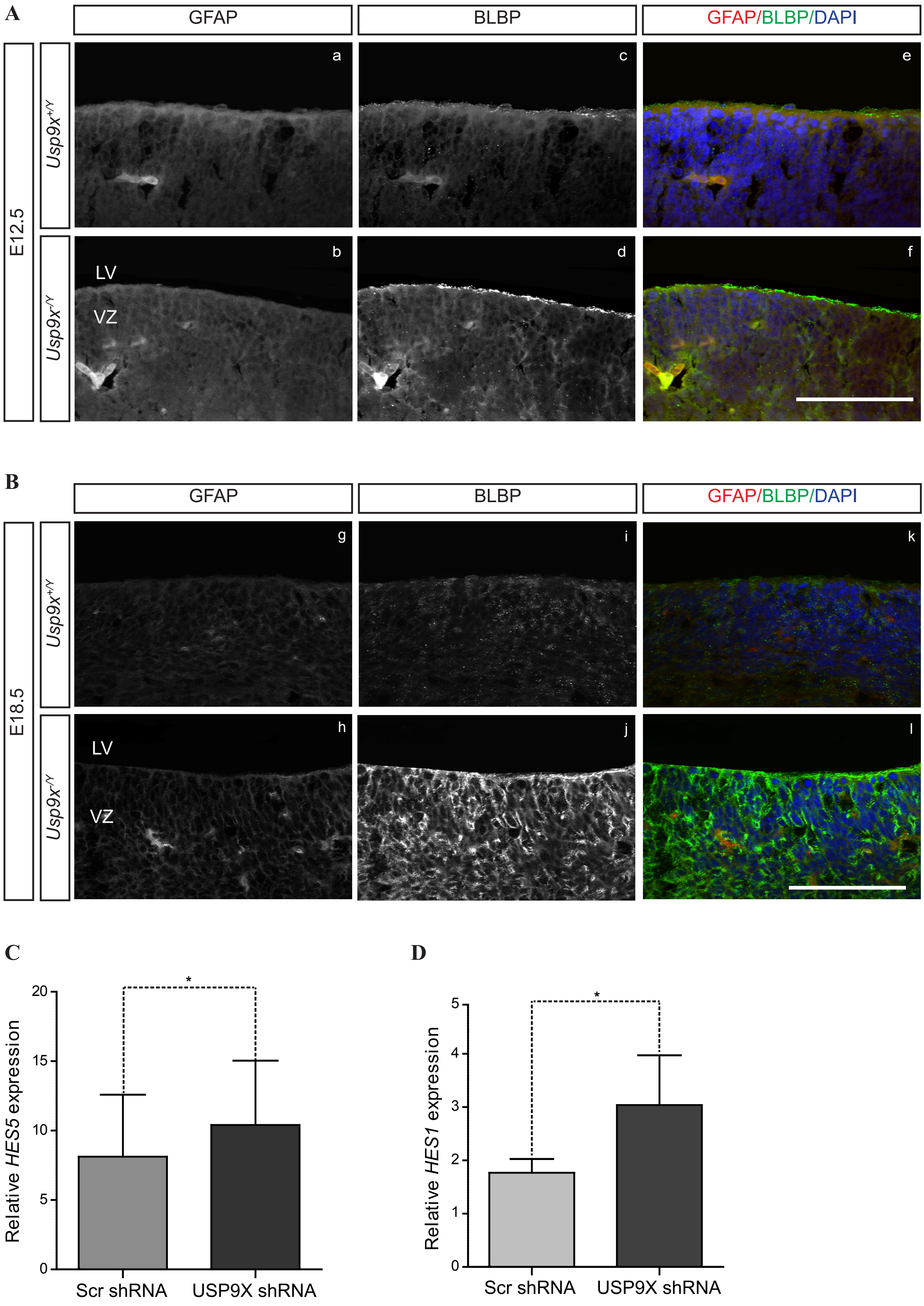

A
GFAP
BLBP
GFAP/BLBP/DAPI
E12.5
Usp9x+/Y
Usp9x-/Y
a
b
c
d
e
f
LV
VZ
B
GFAP
BLBP
GFAP/BLBP/DAPI
E18.5
Usp9x+/Y
Usp9x-/Y
g
h
i
j
k
l
LV
VZ
C
Relative HES5 expression
20
15
10
5
0
*
Scr shRNA
USP9X shRNA
D
Relative HES1 expression
5
4
3
2
1
0
*
Scr shRNA
USP9X shRNA

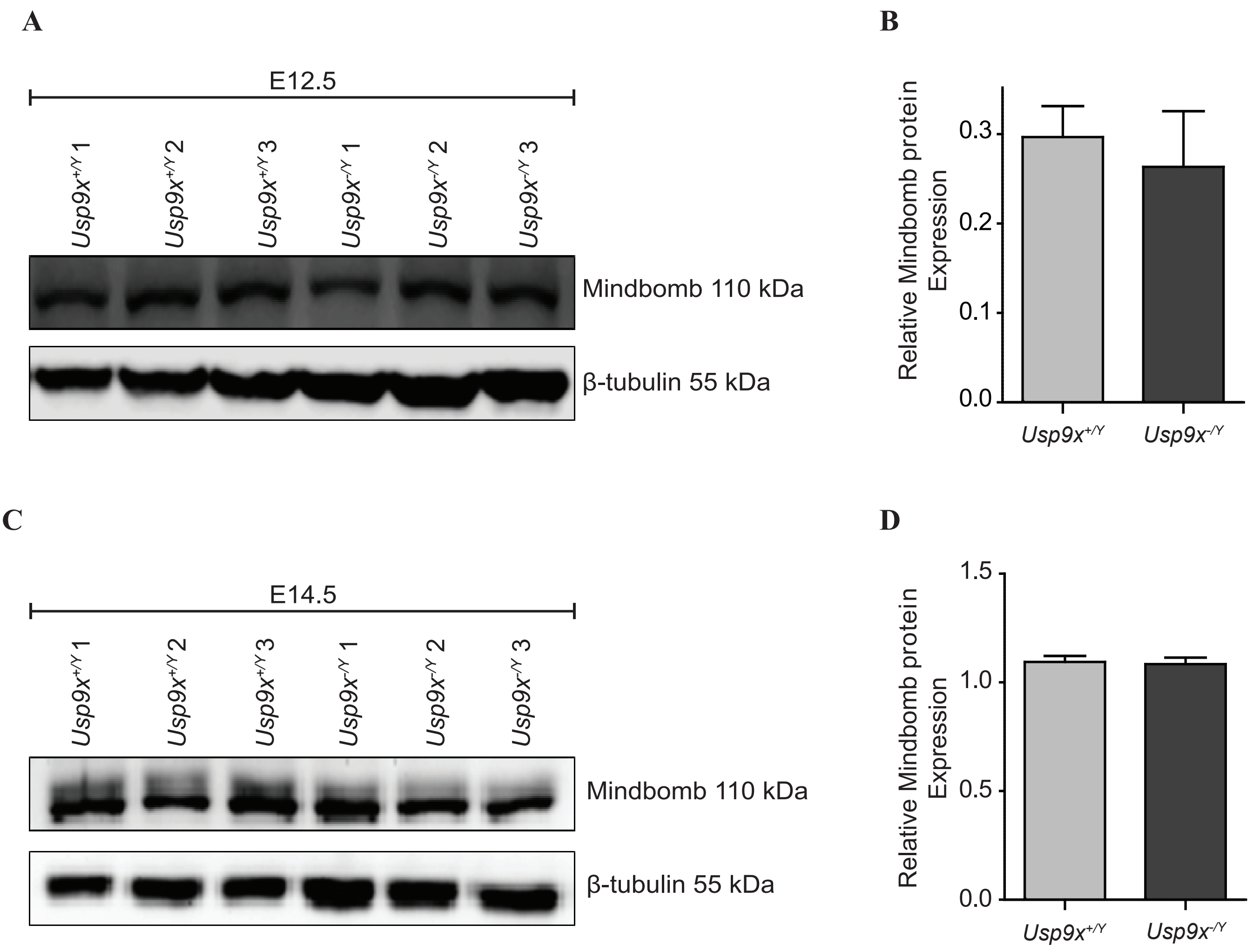
A
E12.5
Usp9x+/Y 1
Usp9x+/Y 2
Usp9x+/Y 3
Usp9x-/Y 1
Usp9x-/Y 2
Usp9x-/Y 3
Mindbomb 110 kDa
β-tubulin 55 kDa
B
Relative Mindbomb protein Expression
0.0
0.1
0.2
0.3
Usp9x+/Y
Usp9x-/Y
C
E14.5
Usp9x+/Y 1
Usp9x+/Y 2
Usp9x+/Y 3
Usp9x-/Y 1
Usp9x-/Y 2
Usp9x-/Y 3
Mindbomb 110 kDa
β-tubulin 55 kDa
D
Relative Mindbomb protein Expression
0.0
0.5
1.0
1.5
Usp9x+/Y
Usp9x-/Y

A

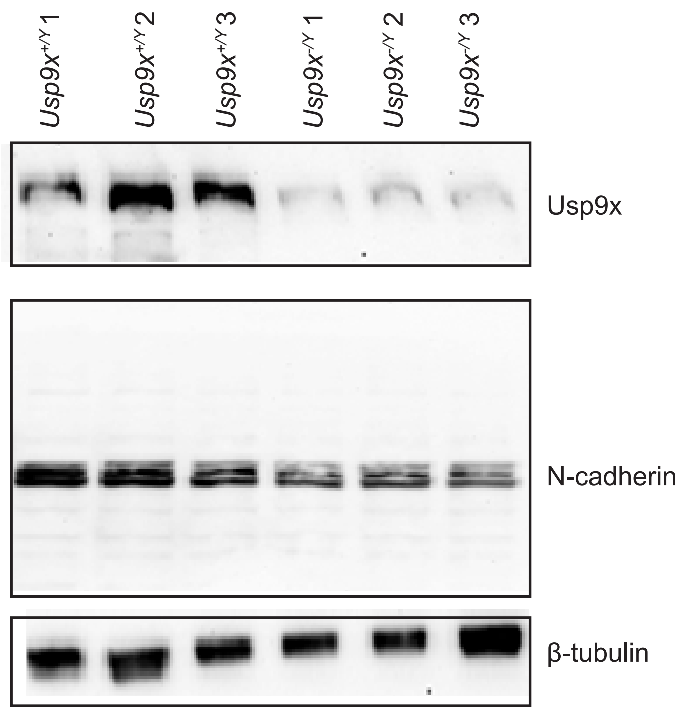

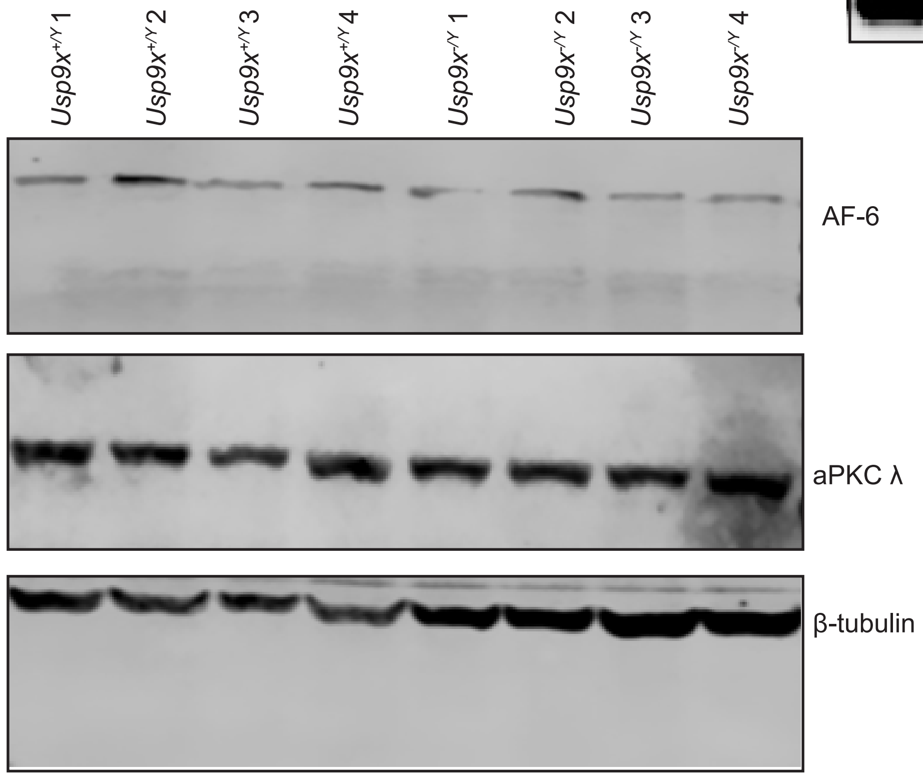

B

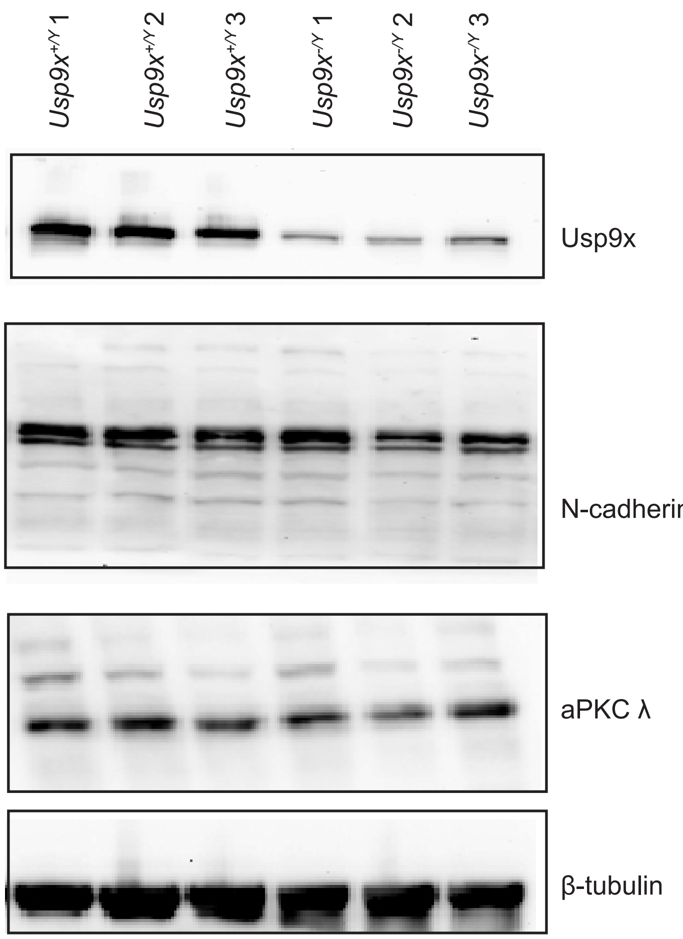

Wood Supplementary Figure 10 to match Figure 1B and 1E full length westernblots

A

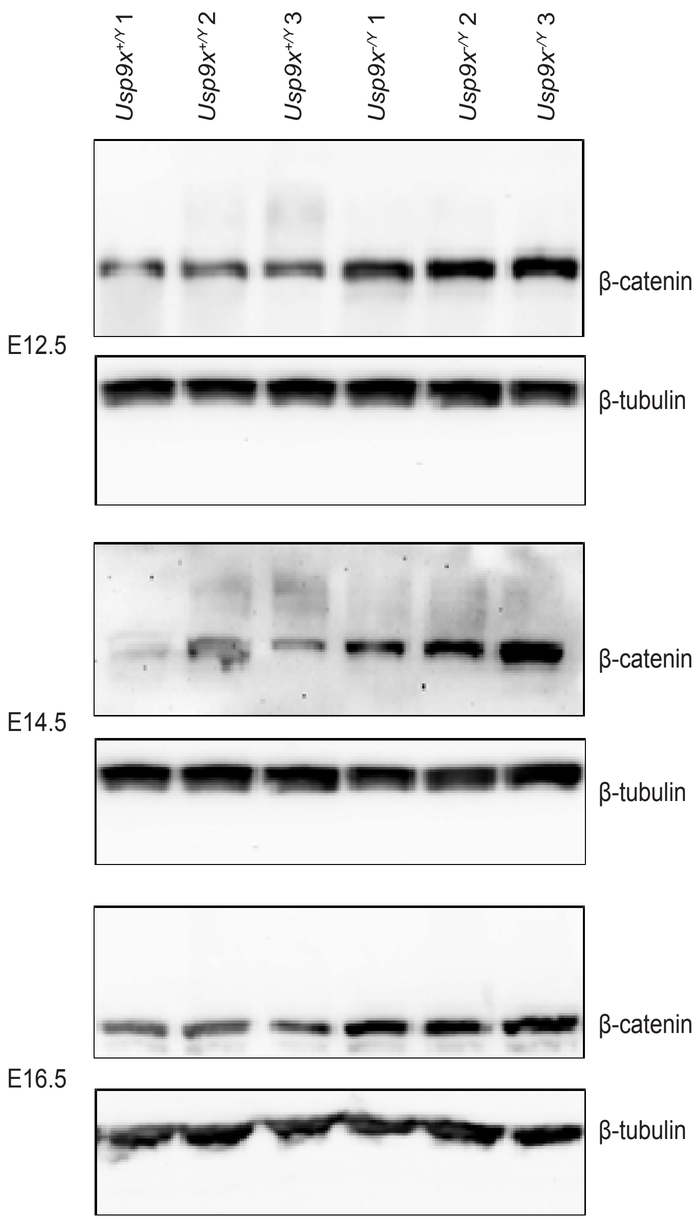

B

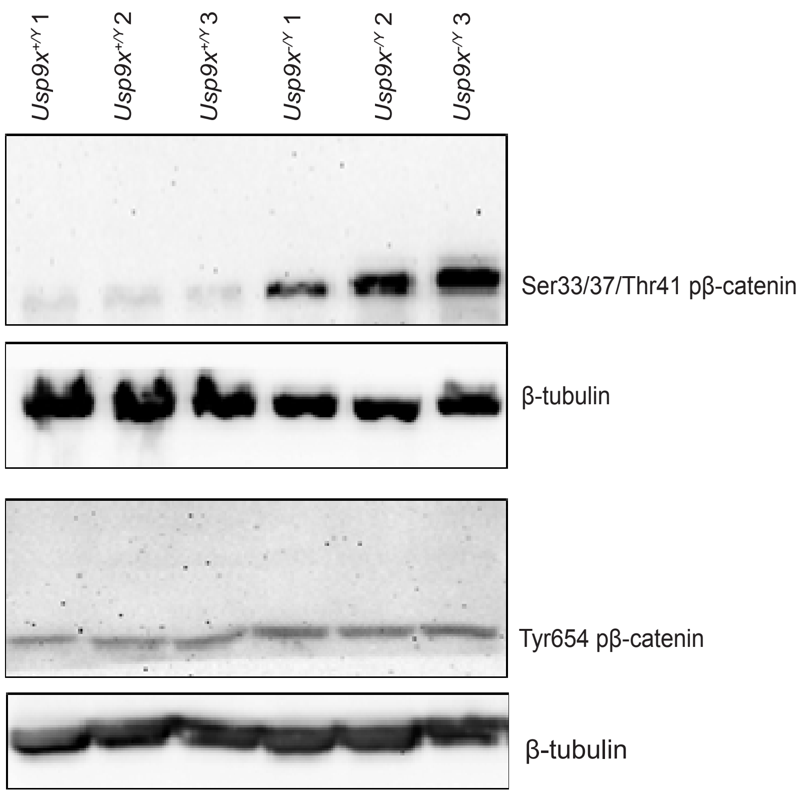

Wood Supplementary Figure 11 to match Figure 3B and 3F full length westernblots

A

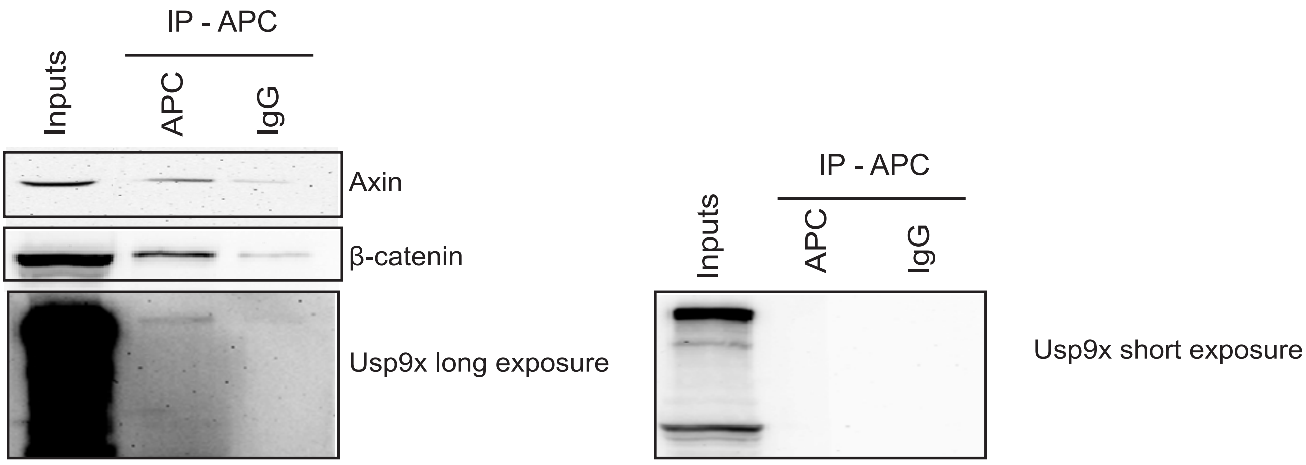

Full length westernblots of Figure 4B

B

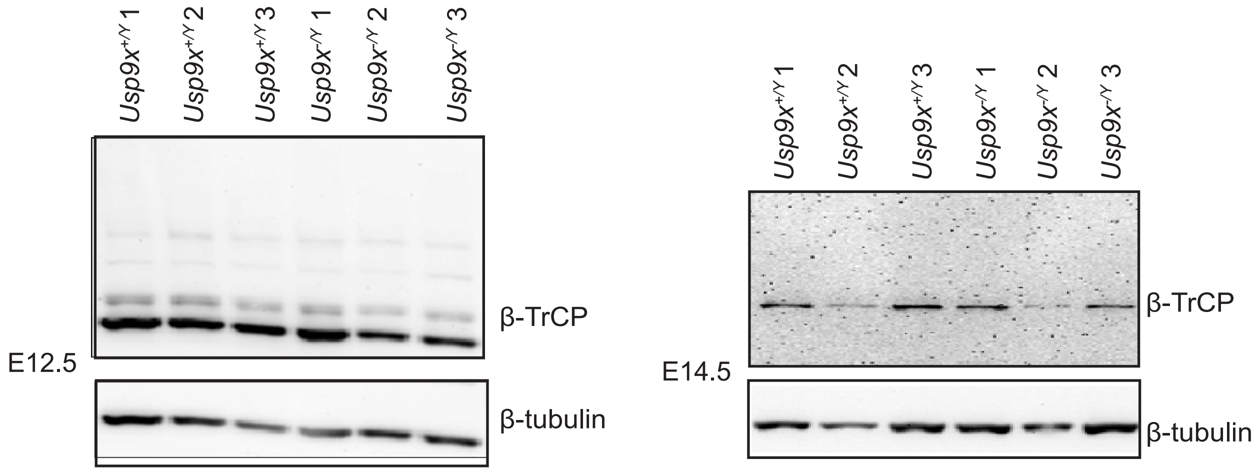

Full length westernblots of Figure 4D

C

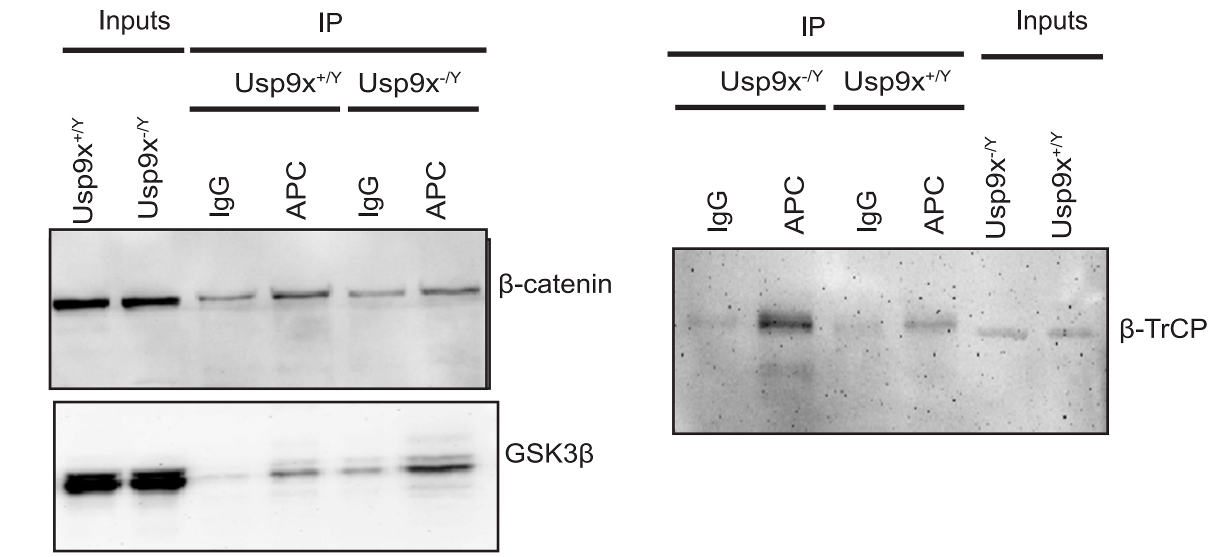

Full length westernblots of Figure 4F

Wood Supplementary Figure 12 to match Figure 4B, F and D full length westernblots

A

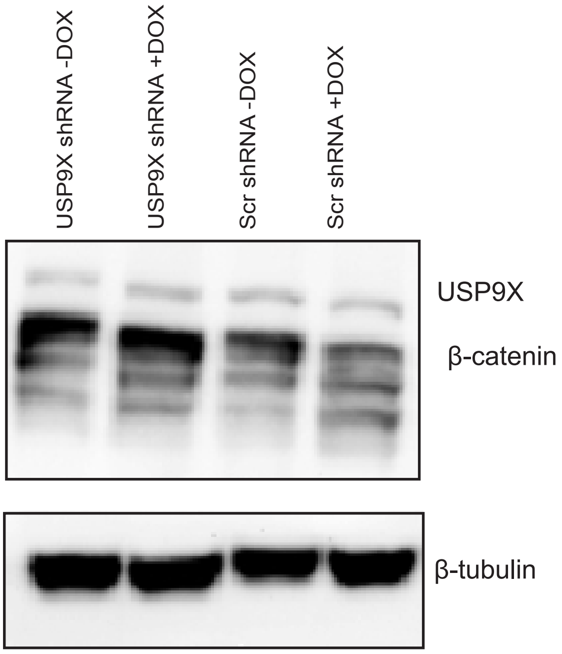

B

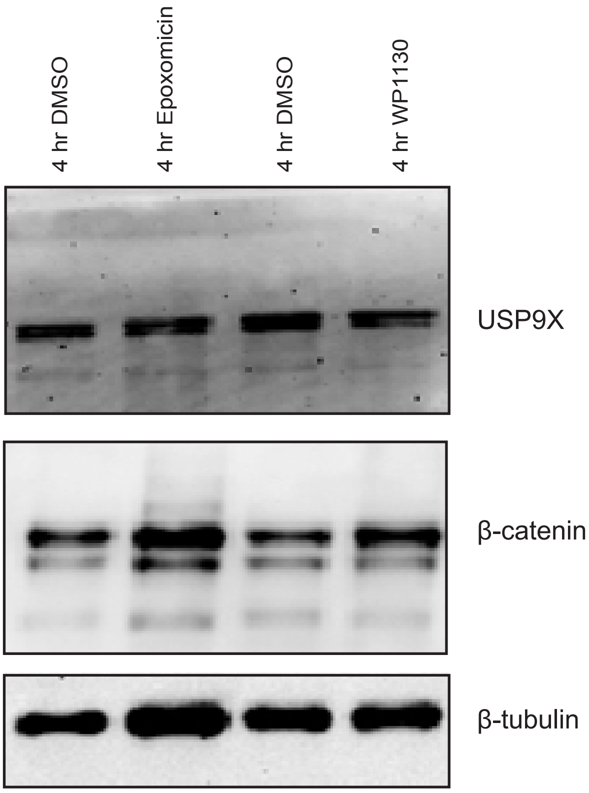

Wood Supplementary Figure 13 to match Figure 5D and Figure 5G full length westernblots

A

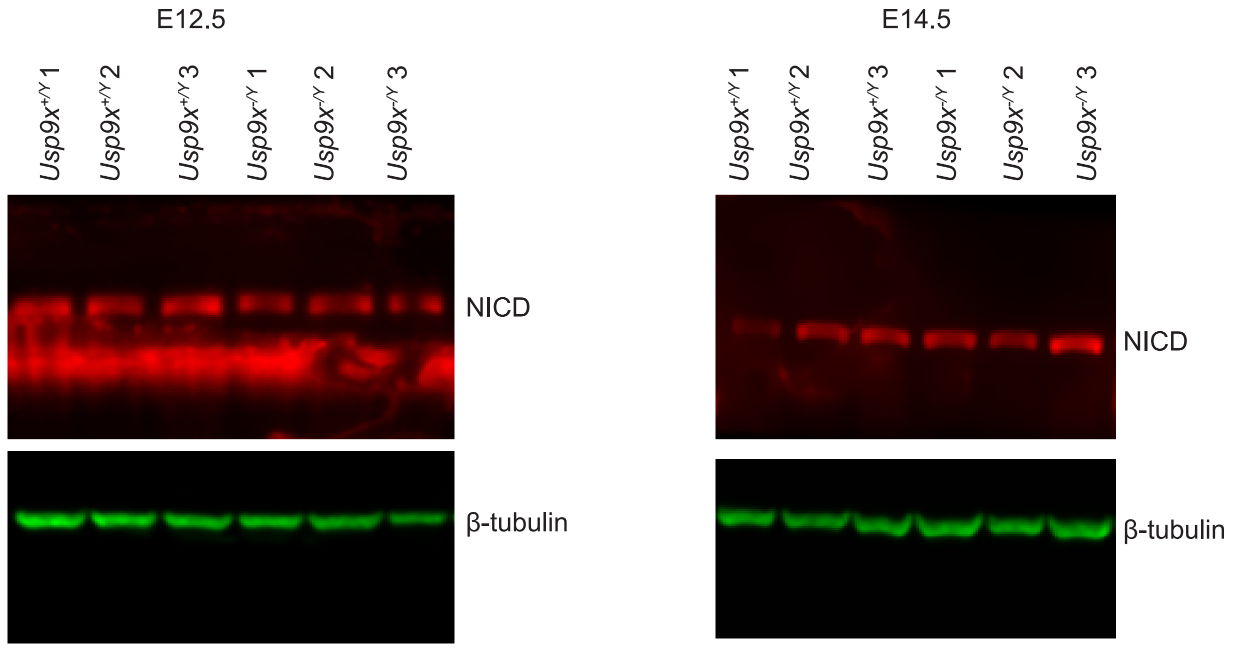

B

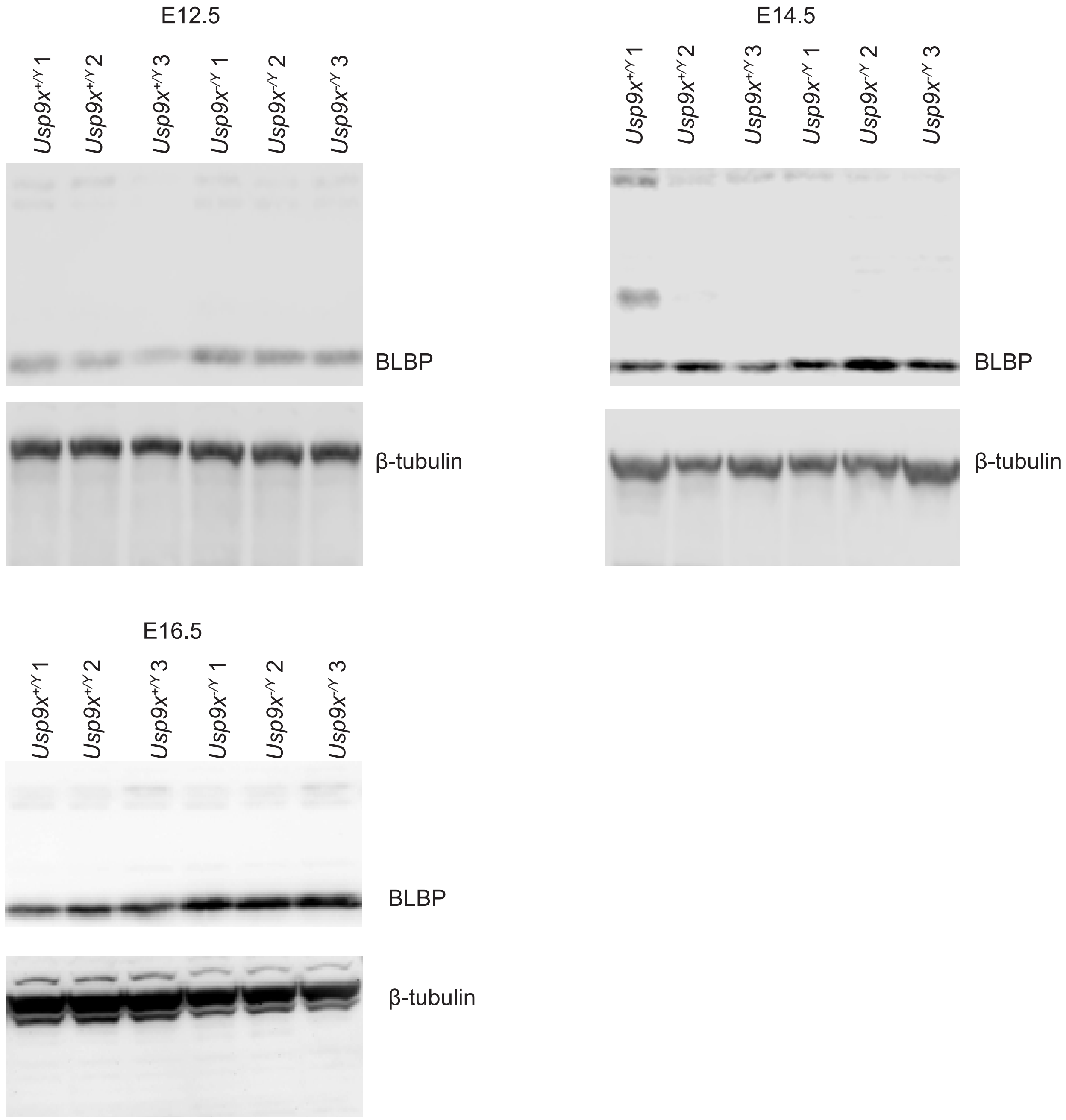

Wood Supplementary Figure 14 to match Figure 6A and Figure 6E full length westernblots

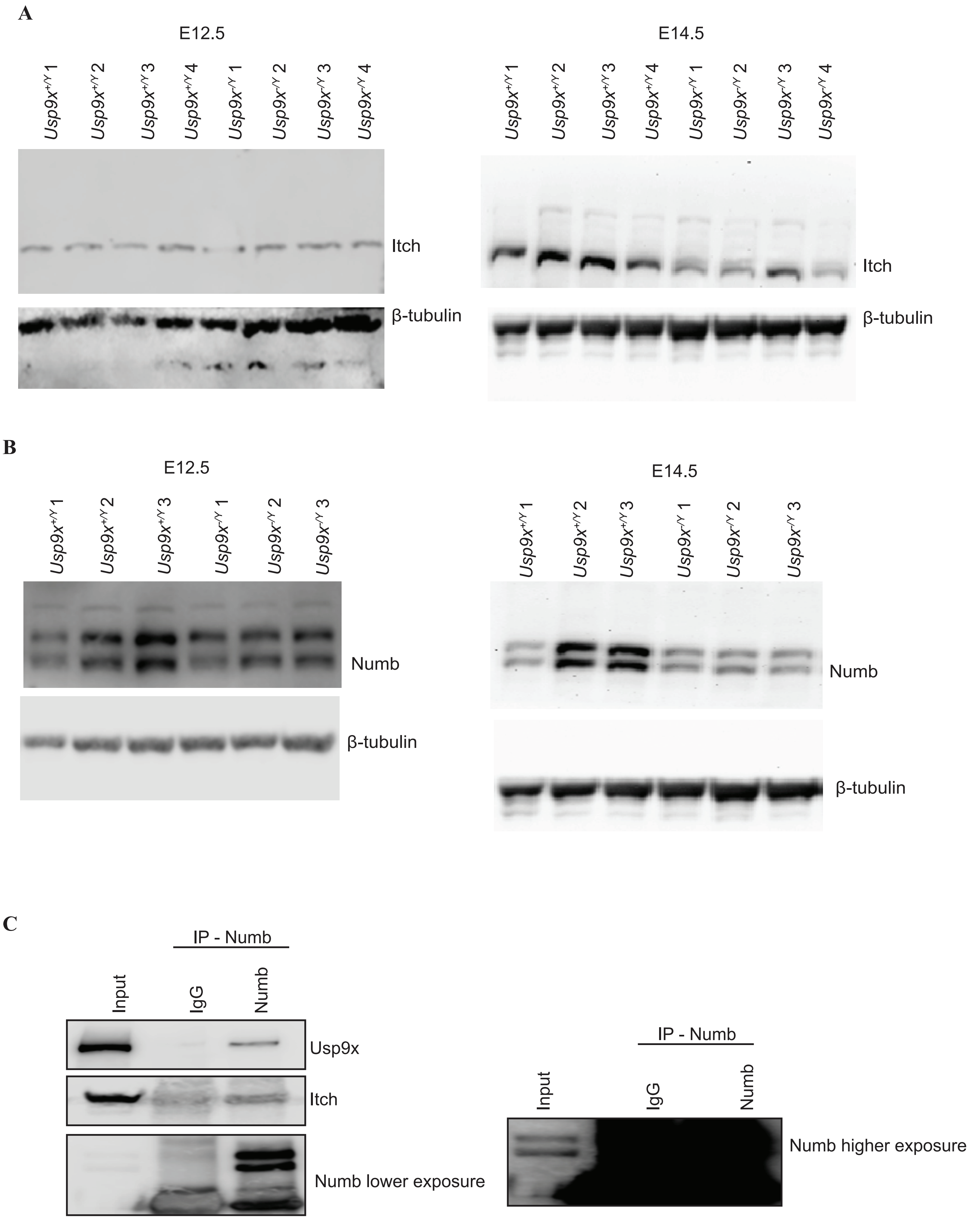

Wood Supplementary Figure 15 to match Figure 7A, D and H full length westernblots
